# Genome‐wide sequence data show no evidence of hybridization and introgression among pollinator wasps associated with a community of Panamanian strangler figs

**DOI:** 10.1111/mec.16373

**Published:** 2022-02-07

**Authors:** Jordan D. Satler, Edward Allen Herre, Tracy A. Heath, Carlos A. Machado, Adalberto Gómez Zúñiga, John D. Nason

**Affiliations:** ^1^ 1177 Department of Ecology, Evolution, and Organismal Biology Iowa State University Ames Iowa USA; ^2^ 56292 Smithsonian Tropical Research Institute Miami Florida USA; ^3^ Department of Biology University of Maryland College Park Maryland USA

**Keywords:** *Ficus*, hybridization, introgression, *Pegoscapus*, phylogeny, pollination mutualism

## Abstract

The specificity of pollinator host choice influences opportunities for reproductive isolation in their host plants. Similarly, host plants can influence opportunities for reproductive isolation in their pollinators. For example, in the fig and fig wasp mutualism, offspring of fig pollinator wasps mate inside the inflorescence that the mothers pollinate. Although often host specific, multiple fig pollinator species are sometimes associated with the same fig species, potentially enabling hybridization between wasp species. Here, we study the 19 pollinator species (*Pegoscapus* spp.) associated with an entire community of 16 Panamanian strangler fig species (*Ficus* subgenus *Urostigma*, section *Americanae*) to determine whether the previously documented history of pollinator host switching and current host sharing predicts genetic admixture among the pollinator species, as has been observed in their host figs. Specifically, we use genome‐wide ultraconserved element (UCE) loci to estimate phylogenetic relationships and test for hybridization and introgression among the pollinator species. In all cases, we recover well‐delimited pollinator species that contain high interspecific divergence. Even among pairs of pollinator species that currently reproduce within syconia of shared host fig species, we found no evidence of hybridization or introgression. This is in contrast to their host figs, where hybridization and introgression have been detected within this community, and more generally, within figs worldwide. Consistent with general patterns recovered among other obligate pollination mutualisms (e.g. yucca moths and yuccas), our results suggest that while hybridization and introgression are processes operating within the host plants, these processes are relatively unimportant within their associated insect pollinators.

## 1 INTRODUCTION

Hybridization between species results in novel genetic combinations derived from divergent parental genomes and can lead to introgression, the transfer of genetic material between species. In many lineages, strong pre‐ and postzygotic barriers limit hybridization and reinforce species boundaries (e.g. Nosil et al., 2005). Evidence from next‐generation sequencing, however, has revealed that hybridization and introgression have occurred throughout the evolutionary history of the tree of life (Mallet et al., 2016; Taylor & Larson, 2019). The influx of genetic material into a lineage introduces new alleles and multilocus combinations that, depending on the situation, can be either harmful (Rhymer & Simberloff, 1996) or (Dowling & Secor, 1997), and thus impact both the genetic diversity and evolutionary dynamics of a species (Payseur & Rieseberg, 2016).

In plant–insect interactions (pollination or herbivorous), higher levels of host specialization generally correspond to a reduction in the diversity of interspecific interactions, potentially limiting opportunities for plant or insect hybridization (de Medeiros & Farrell, 2020). This is particularly true for brood pollination mutualisms (figs and fig wasps, yuccas and yucca moths, leafflowers and leafflower moths) that often exhibit relatively species‐specific host–pollinator relationships (Hembry & Althoff, 2016; Pellmyr et al., 2020; Weiblen, 2002). Therefore, a general question in the evolutionary ecology of host–insect interactions involves identifying the ecological processes that maintain or undermine species boundaries, and generating testable hypotheses concerning factors that affect the evolutionary importance of hybridization and introgression for both host and insect.

Figs (*Ficus*; ca. 800 species) and their obligately associated pollinating wasps (family Agaonidae) present evolutionarily diverse systems that vary in their potential for hybridization. Although most sympatric fig species appear to be pollinated by a single, distinct pollinator fig wasp species, there are many cases where fig species are pollinated by multiple, co‐occurring pollinator species (e.g. Bronstein, 1987; Jackson et al., 2008; Kerdelhué et al., 1997; Machado et al., 2005; Molbo et al., 2004; Ramírez, 1970; Sutton et al., 2017; Wang et al., 2016; Wiebes, 1995a). For example, Yang et al. (2015) found that about 30% of fig species are pollinated by more than one wasp species. When wasps share the same host fig species—especially when different wasp species reproduce inside the same individual inflorescences—there are greater opportunities for hybridization between different pollinator species than when they are the sole pollinator of a host fig.

Fig syconia—urn‐shaped, enclosed inflorescences—define the genus *Ficus*. Chemical, behavioural and morphological traits appear to be important for the female pollinating wasp to identify and locate a receptive fig tree, and enter a receptive fig syconia (Herre et al., 2008). In particular, receptive fig inflorescences produce complex volatile chemical blends recognized by potential pollinators. Generally, the blends produced by different figs are sufficiently distinct for wasps to distinguish among potential hosts (Cornille et al., 2012; Grison‐Pigé et al., 2002; Hossaert‐McKey et al., 2010; Van Noort et al., 1989; Wang, Yang, et al., 2021; Ware et al., 1993). When a female wasp arrives at a fig tree, she must enter a syconium through the ostiole (a small terminal pore) that excludes other insects. Once inside, the female wasp (the foundress) pollinates flowers, oviposits into a subset of them inducing gall formation and dies within the syconium (Janzen, 1979). Offspring then develop over several weeks inside the galled, univolute fig flowers (Galil & Eisikowitch, 1968). Male wasps emerge from their galls, locate galls that contain female wasps and chew holes in the galls to expose females for mating. Importantly, mating among offspring takes place inside the same syconium that was pollinated by the mother. After mating, female pollinators emerge from their galls, gather pollen from male flowers, exit the syconium and disperse. Female wasps routinely travel many kilometres to encounter another receptive fig inflorescence—on typically a conspecific fig tree—to reproduce (Ahmed et al., 2009; Nason et al., 1998).

There are approximately 120 described species of strangler figs (*Ficus* subgenus *Urostigma*, section *Americanae*) in the Neotropics (Berg, 1989). Figs in this group are pollinated by wasps from the genus *Pegoscapus* (family Agaonidae). Cophylogenetic studies of strangler figs and their pollinators—throughout the Neotropics (e.g. Cruaud et al., 2012) and within Panama (e.g. Jackson et al., 2008; Machado et al., 2005; Satler et al., 2019)—have identified highly discordant phylogenetic patterns. Although contemporary associations are predominantly species‐specific, cophylogenetic studies indicate a dynamic evolutionary history punctuated by host‐switching events and pollinators shared between host species, with these events creating opportunities for hybridization among both associated fig and pollinator species.

Pollinator host switching appears to be an important mechanism contributing to hybridization and introgression in the Panamanian strangler fig community (Jackson et al., 2008; Machado et al., 2005). In particular, using genome‐scale data coupled with a model‐based approach, Satler et al. (2019) demonstrated host switching to be the most important process generating phylogenetic patterns in the community of Panamanian strangler figs and pollinating wasps. The demonstration of pollinator sharing and an evolutionary history of host switching by the pollinators associated with the Panamanian strangler figs (Molbo et al., 2003, 2004; Satler et al., 2019) is consistent with observed widespread hybridization and subsequent introgression across most of the Panamanian figs (Jackson et al., 2008; Machado et al., 2005). Further, evidence suggests that hybridization occurs in figs more generally and that introgression is a potentially important process in the evolutionary history of *Ficus* (Bruun‐Lund et al., 2017; Compton, 1990; Compton et al., 2009; Cornille et al., 2012; Renoult et al., 2009; Van Noort et al., 2013; Wang et al., 2016; Wang, Zhang, et al., 2021; Wilde et al., 2020).

While conditions enabling opportunities for fig hybridization only require pollination of a receptive fig by a single wasp carrying heterospecific pollen, conditions for fig wasp hybridization are more rigorous. Given the unusual reproductive biology of the fig and wasp mutualism, if a fig is visited by a single pollinator wasp, all pollinator wasp offspring within the syconium will be her direct descendants—haploid sons and diploid daughters—and mating will be between siblings. In contrast, if two or more foundresses enter and oviposit within the same syconium, there are opportunities for nonsibling mating among their offspring. If these foundresses represent different species, there is the opportunity for hybridization. Since figs vary substantially in their characteristic foundress numbers (Herre, 1985, 1989), opportunities for outcrossing and hybridization in pollinator wasps vary among species. Thus, while fig hybridization only requires pollination by a single wasp carrying heterospecific pollen, to potentially have hybridization and interspecific gene flow between pollinators, multiple foundresses must occur within the same fig syconium, and those foundresses must represent different species.

In addition to evidence of hybridization and introgression in the host strangler figs, hybrid pollinator wasps have been detected between two species within the Panamanian community. For example, Molbo et al. (2003) sampled emerging pollinators from nine fig species. Based on mitochondrial sequence data and microsatellite genotyping, they identified successful F1 hybrids in four of 457 (0.9%) broods sampled from one fig host species, *Ficus obtusifolia*. These F1s occurred between the two frequently co‐occurring wasp species, *Pegoscapus hoffmeyeri* sp. A and *P. hoffmeyeri* sp. B, that locally pollinate *F*. *obtusifolia*. Further, Molbo et al. (2004) recovered two diploid males (out of 18 males sampled from four broods containing hybrid females) among hybrid pollinators associated with *F*. *obtusifolia*. Therefore, although occasional wasp hybridization exists in this system, it is unclear whether successful introgression occurs and how it may affect the evolutionary history of these pollinator wasps. In this study, we address these questions across all pollinators (19 species) associated with the 16 strangler fig species co‐occurring in the Panamanian community using genome‐scale data generated through the application of next‐generation sequencing technologies. This enables both the detection of recent hybridization, and more importantly, quantification of any signals of past introgression, placed within the context of an ecologically and evolutionarily well‐characterized fig wasp community.

The Panamanian strangler fig community is characterized by a history of host switching and contains multiple examples that deviate from the one‐to‐one fig–pollinator association. Because host switching and host sharing have led to hybridization and introgression in the host figs, we ask if these processes have also shaped the evolutionary history of their pollinating wasps. Here, we use genome‐wide sequence data representing ultraconserved element (UCE) loci to estimate phylogenetic relationships and test for hybridization and introgression among a diverse community of fig pollinating wasps. First, we estimate a phylogeny of these wasps and test if individuals sampled from the same host fig species cluster together in phylogenetic space. Next, we test for evidence of hybridization and introgression among five pairs of wasp species for which the opportunities for hybridization appear highest, as they have the potential to pollinate, develop and mate within syconia of the same host fig species. We then test for hybridization and introgression more broadly among all pollinator species. Finally, we consider the ecological and evolutionary processes shaping diversification dynamics in this community of pollinator wasps and how they compare with diversification dynamics in their associated host figs.

## 2 MATERIALS & METHODS

### 2.1 DNA sampling and sequencing

Pollinator wasps were collected from strangler fig species in the vicinity of Barro Colorado Island Nature Monument in the Canal Zone of central Panama (Table 1). Wasps were allowed to emerge from mature figs in the laboratory and then stored in 95% ethanol or RNALater for DNA extraction and analysis. A single wasp was selected per fig infructescence for sequencing to ensure independence among samples. We also included four pollinator wasps from an undescribed *Pegoscapus* species associated with the Mexican strangler fig *Ficus petiolaris* to serve as an outgroup (Cruaud et al., 2012). Including the outgroup, we generated sequence data from 176 wasp samples representing 20 pollinator species, with an average of 8.8 individuals per species (Table 1, Table S1).

**TABLE 1:** Pollinator wasp sampling

Pollinator Wasp	Host Fig	*N*	Foundresses
*Pegoscapus gemellus*	*Ficus bullenei* and *Ficus popenoei*	21	1.980
*Pegoscapus* sp. 1	*Ficus bullenei*	7	1.410
*Pegoscapus* sp. 2	*Ficus popenoei*	11	2.550
*Pegoscapus* sp. 3	*Ficus americana* and *Ficus colubrinae*	16	1.005
*Pegoscapus insularis*	*Ficus americana*	2	1.000
*Pegoscapus orozcoi*	*Ficus colubrinae*	8	1.010
*Pegoscapus hoffmeyeri* sp. A	*Ficus obtusifolia*	4	1.050
*Pegoscapus hoffmeyeri* sp. B	*Ficus obtusifolia*	10	1.050
*Pegoscapus baschierii*	*Ficus turbinata*	8	NA
*Pegoscapus estherae*	*Ficus costaricana*	5	NA
*Pegoscapus grandii*	*Ficus crocata*	17	4.530
*Pegoscapus herrei*	*Ficus paraensis*	12	1.050
*Pegoscapus longiceps*	*Ficus dugandii*	6	2.160
*Pegoscapus lopesi*	*Ficus* aff. *crocata*	10	2.570
*Pegoscapus piceipes*	*Ficus nymphaeifolia*	6	2.640
*Pegoscapus silvestrii*	*Ficus pertusa*	7	NA
*Pegoscapus tonduzi*	*Ficus citrifolia*	6	1.210
*Pegoscapus* sp. 4	*Ficus aurea*	8	NA
*Pegoscapus* sp. 5	*Ficus* sp. 1	8	NA

Note

Information includes pollinator species, host fig species, the number of individual wasps sequenced and the average number of foundresses per host fig.

Foundress information, when present, is from Herre (1989) and represents the average number of foundress wasps per host fig species. An average of 350 (±136) individual figs per fig species were sampled for estimating foundress numbers (see Table 2 in Herre (1989) for details). For figs that share a pollinator species, foundress numbers were averaged between the host figs. Lines separate the three host‐sharing systems from the remaining species (with one‐to‐one fig–wasp association) found in this community.

We used pollinator species names when applicable as described by Wiebes (1995a, 1995b). As several pollinators in this community are undescribed, we denote these species as *Pegoscapus* sp. followed by a unique identifier and their host fig species name. In addition, although a single pollinator species (*P. hoffmeyeri*) was identified morphologically as being associated with *F. obtusifolia* (Wiebes, 1995a), the pollinators comprise two cryptic sister species (Molbo et al., 2003). Consistent with previous studies (Jackson et al., 2008; Molbo et al., 2003, 2004; Satler et al., 2019), we denote these two species as *P. hoffmeyeri* sp. A and *P. hoffmeyeri* sp. B.

Genomic DNA was extracted with a Qiagen DNeasy Kit (Qiagen Inc.). Illumina libraries were generated with a KAPA Hyper Prep kit. Samples were sheared to an average size of ~450 bp on a Covaris sonicator. Following library construction described in Glenn et al. (2019), we grouped samples in sets of eight and hybridized biotinylated RNA probes to capture targeted loci. We used the hymenopteran probe v2 set of Branstetter et al. (2017) to target 2590 UCE loci. After probe hybridization and library amplification, size distributions were checked on a Bioanalyzer and libraries were combined in equimolar concentrations for sequencing. Libraries were sequenced on an Illumina sequencer targeting 150 bp paired‐end reads.

### 2.2 Data processing

We used Phyluce v1.6.7 (Faircloth, 2015) to process raw sequence reads and generate data sets for downstream analysis. Sequence reads were cleaned with illumiprocessor v2.0.9 (Faircloth, 2013), a wrapper around Trimmomatic v0.39 (Bolger et al., 2014). Cleaned reads were assembled into contigs with Trinity v2.0.6 (Grabherr et al., 2011). Contigs were then aligned to the hymenopteran v2 UCE locus set to filter nonspecific sequences. Loci were subsequently aligned with MAFFT v7.407 (Katoh & Standley, 2013), and ambiguously aligned sites were removed with Gblocks v0.91b (Castresana, 2000) using default parameters. All loci sampled for a minimum of 70% of individuals were retained in the final data set. Additionally, we generated a data set of phased alleles for certain downstream analyses following the outline provided by Andermann et al. (2018). For this, we took our aligned loci (before the use of Gblocks) and mapped cleaned sequence reads for each individual to the UCE locus set using BWA‐MEM (Li, 2013) in bwa v0.7.17 (Li & Durbin, 2010). We then phased the data with samtools v1.9 (Li et al., 2009) using the phase command, calling two alleles per individual per locus. Loci were then realigned and cleaned as described above, with loci sampled for a minimum of 70% of individuals retained for downstream analysis.

### 2.3 Phylogenetics

We used both concatenation and coalescent‐based species tree methods to estimate phylogenetic relationships among the fig wasps. We first estimated a concatenated phylogeny using maximum likelihood (ML) in IQ‐TREE v1.6.12 (Chernomor et al., 2016; Nguyen et al., 2015). This approach allows us to test species monophyly as individuals are treated as tips in the tree. The data set was partitioned by UCE locus, with each partition estimated under the GTR + γ substitution model. Nodal support values were generated through 1000 repetitions of the ultrafast bootstrap approximation (Hoang et al., 2018).

We used two coalescent‐based approaches to estimate a species tree. Rather than assuming all genes evolved under the same tree topology, these methods allow for discordance between the gene histories and species tree by explicitly accounting for the biological process of incomplete lineage sorting. First, we used the program SVDQuartets (Chifman & Kubatko, 2014) as implemented in PAUP* v4.0a166 (Swofford, 2003). SVDQuartets uses site patterns in the nucleotide data to estimate a phylogeny under the multispecies coalescent model. We used SVDQuartets in two ways. We initially estimated a lineage tree (SVDQ_LT_), where all individuals are represented as tips in the tree, to confirm species assignment and test species monophyly. We then assigned individuals to species a priori and estimated a species tree (SVDQ_ST_). For both SVDQuartets analyses, we evaluated all quartets and used standard bootstrapping to generate nodal support values. Second, we used the program ASTRAL‐III v5.6.3 (Zhang et al., 2018) to estimate a species tree. Instead of using site patterns in the nucleotide data, ASTRAL‐III uses gene trees as input to estimate a species tree. Maximum‐likelihood gene trees were first estimated in IQ‐TREE. For each locus, IQ‐TREE selected the substitution model of best fit with ModelFinder (Kalyaanamoorthy et al., 2017) using Bayesian Information Criteria (BIC). We assigned individuals to species a priori (Rabiee et al., 2019) and then used the ML gene trees as input to estimate a species tree. Nodal support values were quantified with local posterior probability values (Sayyari & Mirarab, 2016).

Previous work in the Panamanian system has recovered pollinator species as monophyletic and has not brought into question species validity (Jackson et al., 2008; Machado et al., 2005; Satler et al., 2019). Given the typical pattern of small intraspecific divergence coupled with large interspecific divergence for these wasps, we wanted to ask what proportion of loci recover each species as monophyletic. If the pollinators have been evolving in isolation over evolutionary time, we would expect the species to be monophyletic for all or nearly all sampled loci. In contrast, if interspecific gene flow has been an important process in the evolution of this system, we would expect interacting species to show a lack of monophyly for numerous loci. We used DendroPy v4.4.0 (Sukumaran & Holder, 2010) to count the proportion of gene trees (as estimated above in IQ‐TREE) for which a species was monophyletic. We required that at least two individuals were sequenced for a species for a given gene tree to assess monophyly.

### 2.4 Population genetics

In addition to estimating phylogenetic relationships and testing for monophyly, we were interested in understanding the population genetics of the pollinator species. In particular, we wanted to test whether genetic diversity within a pollinator species varied with average foundress number per host fig. If outbreeding is common in pollinator species that contain multiple foundresses per fruit, we would expect to see a positive correlation between genetic diversity and average foundress number. We used DendroPy to calculate nucleotide diversity (*π*), number of segregating sites and Watterson's theta (per site) for all pollinator species. For species with foundress number data (see Table 1), we used Spearman's rank correlation test in r v3.5.2 (R Core Team, 2018) to test for a correlation between average number of foundresses and summary statistic.

### 2.5 Testing for monophyly with mitochondrial DNA

To complement analyses and inference with the genome‐wide sequence data from the nuclear genome, we used mitochondrial DNA (mtDNA) to test whether pollinator species were monophyletic in the mitochondrial genome. Even when not targeted, mitochondrial DNA is often collected using sequence capture approaches (Barrow et al., 2017; do Amaral et al., 2015). We used NOVOPlasty v3.8.3 (Dierckxsens et al., 2017) to identify mitochondrial reads and generate haplotypes from the sequencing files. Since no *Pegoscapus* mitochondrial reference genome is available, we used a *P. hoffmeyeri* (AY148119) cytochrome oxidase I (COI) mtDNA sequence as a seed with which to align the reads. We used default settings with an initial kmer value of 39. For samples that either did not generate any matches or produced obvious sequencing errors, we lowered the kmer value to 23. Using a lower kmer value can help with low coverage data, appropriate here since we were only targeting regions of the nuclear genome. Finally, for samples that did not recover any mtDNA with the initial seed sequence, we aligned reads (using default settings) to a longer COI sequence of a different pollinator species (*Pegoscapus* sp., JN103329) to see whether a different seed sequence could recover mtDNA data.

We aligned the mitochondrial sequences with MAFFT and edge trimmed to match the primary seed sequence (AY148119) to reduce missing data. We used DendroPy to calculate several summary statistics (as described above) to characterize genetic variation within species. In addition, we used PAUP* to calculate GTR corrected genetic distances for both within‐species and between‐species comparisons. Finally, we estimated a maximum‐likelihood gene tree in IQ‐TREE, using ModelFinder to select the substitution model and 1000 repetitions of the ultrafast bootstrap approximation to generate nodal support. If patterns of monophyly differ between the nuclear and mitochondrial genomes, the cytonuclear discordance would suggest introgression or processes other than genetic drift generating these discordant patterns.

### 2.6 Testing for admixture and gene flow

**FIGURE 1:**
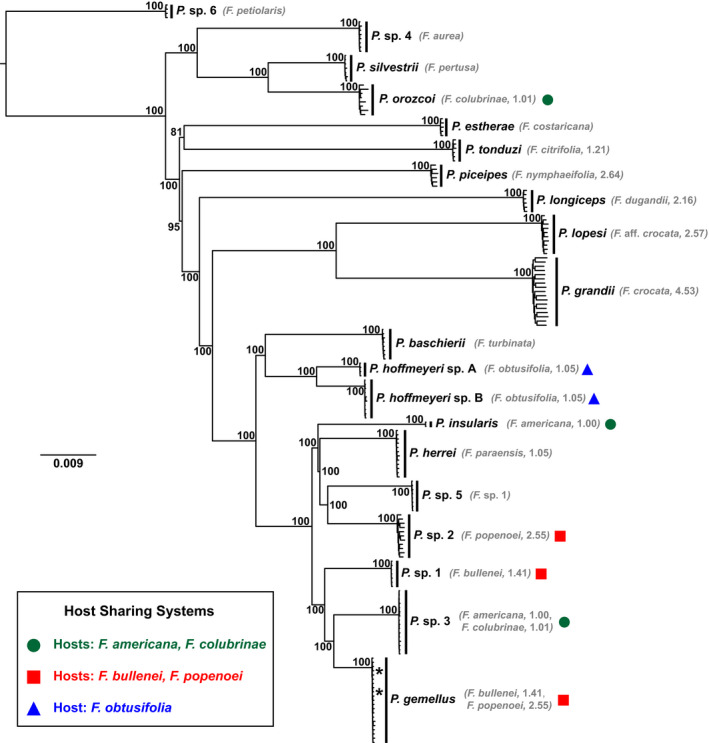
Maximum‐likelihood phylogeny representing relationships among *Pegoscapus* wasps. Host fig species are displayed next to their associated wasp species, with average foundress number data included when available. See Herre (1989) for details. Node labels represent bootstrap support values. The insert shows the three systems with co‐occurring pollinators, denoted by a circle (green), square (red) or triangle (blue). Two individuals (represented by asterisks) of *Pegoscapus gemellus* (hosts: *Ficus bullenei*/*Ficus popenoei*) were sampled from *Ficus dugandii*, not its normal host. The undescribed pollinator species associated with *Ficus petiolaris* was used to root the tree

Detailed studies of Neotropical strangler figs and their pollinators have revealed exceptions to the one‐to‐one association between fig and wasp species. Specifically, cases in which multiple different wasp species (based on COI and microsatellite loci) pollinate a single host fig, and some cases where the same wasp species pollinates more than one host, have been found (Machado et al., 2005; Molbo et al., 2003, 2004). Cases of host sharing, where multiple pollinator species are associated with the same host, are of particular interest because they present clear opportunities for hybridization and introgression among these different wasp species, which range in their phylogenetic similarity from being closely related to being distantly related (Satler et al., 2019). Five cases of host sharing have been described in central Panama: (i and ii) *Ficus americana* and *Ficus colubrinae* share a pollinator, *Pegoscapus* sp. 3, which co‐occurs with *Pegoscapus insularis* in *F*. *americana* and *Pegoscapus orozcoi* in *F*. *colubrinae*, resulting in two hosts each with two co‐occurring pollinators, and (iii and iv) *Ficus bullenei* and *Ficus popenoei* share a pollinator, *Pegoscapus gemellus*, which co‐occurs with *Pegoscapus* sp. 1 in *F*. *bullenei* and *Pegoscapus* sp. 2 in *F*. *popenoei*, again resulting in two hosts each with two co‐occurring pollinators, and (v) *F. obtusifolia* is pollinated by two co‐occurring species, *P. hoffmeyeri* sp. A and *P. hoffmeyeri* sp. B. We refer to these three systems as AC (i and ii), BP (iii and iv) and O (v) (see Figure 1). They include two pollinator species associated with two hosts each, and six pollinator species that are host specific. It is the interaction of wasps within the same fig hosts that provide the greatest opportunities for hybridization and gene flow.

The five fig species with co‐occurring pollinator species vary in their average number of foundress wasps per fig (Herre, 1989). Variation in foundress number enables predictions about the pollinator taxa most likely to contain histories of hybridization and gene flow. *Ficus americana* and *F*. *colubrinae* are nearly always single foundress (Table 1). Despite two pollinator species associated with each fig species, we expect little opportunity for hybridization since more than one foundress rarely occupies the same syconium. *Ficus obtusifolia* is mostly single foundress, averaging 1.21 foundresses per fruit, although 16% of sampled fruits had between two and four foundresses (Herre, 1989). We expect intermediate opportunities for hybridization between the two pollinators associated with this host. Finally, *F*. *bullenei* averages 1.41 foundresses per fruit and *F*. *popenoei* averages 2.55 foundresses per fruit, providing frequent opportunities for pollinator co‐occurrence and, potentially, hybridization between their two pairs of wasp species. Together, the five pairs of host‐sharing pollinators vary substantially in their expected opportunities for hybridization and interspecific gene flow.

We used two approaches to test for hybridization and admixture between co‐occurring pollinator species. First, we used principal components analysis (PCA). If sampled individuals are recent hybrids or backcrosses to parental lineages, we would expect to see individuals intermediate between clusters representing parental lineages. Further, if introgression has been extensive, there should be little separation between these clusters in PCA space. PCA was conducted with the dudi.pca function in the r package ade4 dray (Dray & Dufour, 2007). Missing data were replaced with mean values of allele frequencies within a given species. We plotted the first two axes of variation to visualize genetic clustering of individuals. Second, we used Structure v2.3.4 (Pritchard et al., 2000) to cluster individuals into genetic groupings. Structure clusters individuals by maximizing Hardy–Weinberg equilibrium within clusters and minimizing Hardy–Weinberg equilibrium among clusters. Evidence for recent hybridization and introgression would be reflected by individuals containing multilocus genotypes sampled from multiple clusters. Our choice of the number of clusters (*K*) was informed by previous work identifying the wasp species within these systems (Jackson et al., 2008; Molbo et al., 2003). For the *F*. *americana*/*F*. *colubrinae* (AC) and *F*. *bullenei*/*F*. *popenoei* (BP) host sharing systems, we used a *K* value of 3; for *F*. *obtusifolia* (O), we used a *K* value of 2. We used the admixture model and allowed allele frequencies to be correlated among populations. For each analysis, we used a burnin of 100,000 steps, followed by 500,000 MCMC reps, and completed 10 replicates. We processed results in the r package pophelper v2.2.7 (Francis, 2017).

Genetic data were processed independently for pollinators in each host sharing system to generate phased data sets. By processing each system independently, we retained loci with a minimum of 70% of individuals for the taxa of interest only. We followed the same procedure as outlined above for generating the phased data sets and used unlinked SNPs for both the PCA and Structure analyses. To generate these data sets, we first scanned our phased aligned taxon‐specific UCE loci for variable sites. Within a locus, we then selected the SNP that had the highest sample coverage. If a locus had multiple SNPs with equal sample coverage, one of those SNPs was selected at random.

Tests of hybridization among co‐pollinators provide biologically motivated hypotheses where opportunities for interspecific mating are most likely. If gene flow among these species has been recent or ongoing, we would expect to detect that signal and infer a history of hybridization. This approach, however, is limited to specific sets of taxa based on present‐day associations. Given the history of host switching in this system, and accounting for a potential dynamic process of host–pollinator associations through time, it would be useful to test for hybridization among all sampled taxa.

To test whether historical introgression has been an important process in this community of pollinators, we used TreeMix v1.13 (Pickrell & Pritchard, 2012). TreeMix is a maximum‐likelihood approach that uses allele frequency data to construct a population graph and places hybridization events on that graph for populations with the least fit to a tree model. Since the number of hybridization events is specified a priori, we can test models varying the number of hybridization events to determine a model of best fit. We used the total variance explained by the model to inform the number of hybridization events that best characterizes this community of pollinator species. We used allele frequency data representing unlinked, biallelic SNPs from the phased data set from all pollinator species. To remove effects of missing data, we first identified biallelic SNPs within each locus that were sampled for at least one individual per species. For loci with multiple SNPs, we selected a single SNP with the highest coverage. If a locus had multiple SNPs with equal sample coverage, one SNP was selected at random. We then estimated a maximum‐likelihood population graph in TreeMix, allowing between zero and five migration events.

Although additional approaches are often used to test for hybridization and introgression, they were not applicable for our data set. For example, *D*‐statistics (Durand et al., 2011; Green et al., 2010), often referred to as ABBA/BABA tests, have been widely used to infer a signal of introgression among numerous clades across the tree of life (e.g. Eaton & Ree, 2013; Hughes et al., 2020; Meier et al., 2017; Pulido‐Santacruz et al., 2020; Streicher et al., 2014). *D*‐statistics, however, cannot estimate gene flow between sister species, and our biologically informed hypotheses do not lend themselves well to the strict phylogenetic pattern (((P1,P2),P3),OG) typically required for testing if one species (P3) has hybridized with another (either species P1 or species P2). In addition, if we use an agnostic approach and test all possible combinations of taxa, that would require 969 tests (19 choose 3) and would potentially lead to artefacts from unsampled (‘ghost’) lineages given the subsampling approach (Pease & Hahn, 2015).

## 3 RESULTS

### 3.1 DNA sampling and sequencing

We generated 468,733,333 total sequence reads from the 176 fig wasp samples, resulting in an average of 2,663,258 (±1,273,010) reads per individual. Assembly in Trinity resulted in an average of 139,295 (±80,671) contigs per individual. After filtering and retaining contigs that matched UCEs in the probe set, we retained an average of 1590 (±179) UCE loci per individual. Filtering loci for those that contained at least 70% of individuals sampled, we retained 1504 loci for the complete taxonomic data set. In the three systems (processed independently) with shared and unique pollinators, at 70% sequencing threshold, we retained 1388 (AC), 1438 (BP), and 1390 (O) loci for analysis.

### 3.2 Phylogenetics

When individuals are treated as tips in the tree, phylogenetic analyses recover each pollinator species as monophyletic with strong support. We find this result with both concatenation (Figure 1) and the coalescent‐based lineage tree analysis with SVDQuartets (SVDQ_LT_, Figure S1). To further investigate, we tested species monophyly for each sampled UCE locus. All species were monophyletic for all or nearly all loci (Table 2). 58% of species were monophyletic in at least 95% of loci, while 84% of species were monophyletic in at least 90% of loci. Only three species were monophyletic at fewer than 90% of their loci, with the lowest proportion being 82% (*P*. *gemellus* [hosts: *F*. *bullenei*/*F*.* popenoei*]). For species with foundress data (see Table 1), there is no significant correlation between average foundress number and proportion of monophyletic loci (Spearman's rank correlation test, *ρ* = .280, *p* = .333). Although we do not distinguish the causes of nonmonophyly (deep coalescence, introgression and gene tree estimation error), the estimates of monophyly are very high and provide strong support for the validity of the wasp species and their isolation over evolutionary time.

**TABLE 2:** Population genetic summary statistics for the pollinator wasp species

Pollinator Wasp	Host Fig	*π*	SS	*θ_w_*	Monophyly
*Pegoscapus gemellus*	*Ficus bullenei* and *Ficus popenoei*	0.0005 (±0.0014)	2.0410 (±2.9494)	0.0012 (±0.0018)	0.82 (1502)
*Pegoscapus* sp. 1	*Ficus bullenei*	0.0005 (±0.0025)	0.5179 (±1.4514)	0.0005 (±0.0018)	0.92 (1482)
*Pegoscapus* sp. 2	*Ficus popenoei*	0.0013 (±0.0021)	2.4979 (±3.4233)	0.0016 (±0.0022)	0.89 (1496)
*Pegoscapus* sp. 3	*Ficus americana* and *Ficus colubrinae*	0.0004 (±0.0018)	0.8855 (±2.4233)	0.0007 (±0.0020)	0.91 (1499)
*Pegoscapus insularis*	*Ficus americana*	0.0003 (±0.0032)	0.1600 (±1.5342)	0.0003 (±0.0026)	0.98 (1337)
*Pegoscapus orozcoi*	*Ficus colubrinae*	0.0024 (±0.0135)	2.3022 (±2.6740)	0.0021 (±0.0025)	0.94 (1484)
*Pegoscapus hoffmeyeri* sp. A	*Ficus obtusifolia*	0.0004 (±0.0022)	0.3750 (±1.7908)	0.0004 (±0.0021)	0.93 (1479)
*Pegoscapus hoffmeyeri* sp. B	*Ficus obtusifolia*	0.0004 (±0.0026)	0.6432 (±1.7933)	0.0005 (±0.0018)	0.87 (1493)
*Pegoscapus baschierii*	*Ficus turbinata*	0.0005 (±0.0024)	0.6733 (±1.6915)	0.0006 (±0.0015)	0.95 (1484)
*Pegoscapus estherae*	*Ficus costaricana*	0.0007 (±0.0022)	0.8545 (±2.5590)	0.0008 (±0.0022)	0.99 (1467)
*Pegoscapus grandii*	*Ficus crocata*	0.0029 (±0.0029)	8.1135 (±7.9945)	0.0047 (±0.0040)	0.96 (1501)
*Pegoscapus herrei*	*Ficus paraensis*	0.0008 (±0.0025)	1.4675 (±2.4902)	0.0011 (±0.0025)	0.91 (1499)
*Pegoscapus longiceps*	*Ficus dugandii*	0.0009 (±0.0026)	1.0470 (±2.3325)	0.0009 (±0.0023)	1.00 (1472)
*Pegoscapus lopesi*	*Ficus* aff. *crocata*	0.0013 (±0.0027)	2.1351 (±3.5987)	0.0015 (±0.0024)	0.99 (1484)
*Pegoscapus piceipes*	*Ficus nymphaeifolia*	0.0015 (±0.0030)	1.8930 (±3.1201)	0.0015 (±0.0026)	0.99 (1476)
*Pegoscapus silvestrii*	*Ficus pertusa*	0.0004 (±0.0016)	0.4931 (±1.7470)	0.0004 (±0.0017)	0.97 (1488)
*Pegoscapus tonduzi*	*Ficus citrifolia*	0.0006 (±0.0025)	0.7403 (±2.2139)	0.0006 (±0.0019)	0.99 (1467)
*Pegoscapus* sp. 4	*Ficus aurea*	0.0005 (±0.0015)	0.7237 (±2.0046)	0.0006 (±0.0019)	0.99 (1490)
*Pegoscapus* sp. 5	*Ficus* sp. 1	0.0003 (±0.0013)	0.4135 (±1.4957)	0.0004 (±0.0015)	0.96 (1491)

Note

Summary statistics include nucleotide diversity (*π*), number of segregating sites (SS) and Watterson's theta per site (*θ_w_*). Monophyly shows the proportion of gene trees for which a pollinator species is monophyletic. A gene tree was tested for monophyly when two or more sequences for a given species were sampled, with total trees tested per species in parentheses. Lines separate the three host‐sharing systems from the remaining species (with one‐to‐one fig–wasp association) found in this community.

**FIGURE 2:**
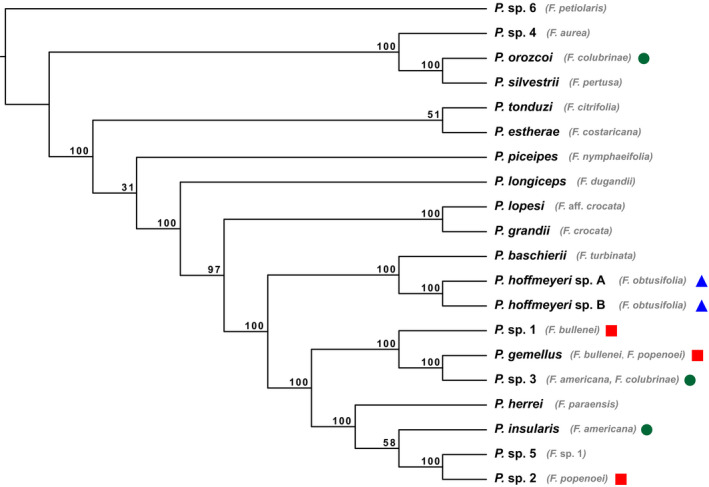
Species tree analysis with SVDQuartets (SVDQ_ST_). Nodal support is denoted with bootstrap support values. Host fig species are displayed next to their respective wasp species. Co‐occurring pollinators are denoted by a circle (green), square (red) or triangle (blue). The undescribed pollinator species associated with *Ficus petiolaris* was used to root the tree

Phylogenetic relationships are congruent across all three methods. For the concatenated tree, all nodes are strongly supported (most have a bootstrap value of 100), with a general pattern of small intraspecific divergence and large interspecific divergence (Figure 1). Two *P*. *gemellus* (hosts: *F*. *bullenei*/*F*. *popenoei*) individuals were sampled from *Ficus dugandii*, indicating an example of nonhost specificity. In the species tree analyses SVDQ_ST_ (Figure 2) and ASTRAL‐III (Figure S2), there is strong support for most relationships among species, although a few nodes in both trees are weakly supported. The minor differences in the phylogenetic analyses occur at nodes with low support or at short internal branches. For example, IQ‐TREE supports *Pegoscapus herrei* (host: *Ficus paraensis*) sister to (*Pegoscapus* sp. 5 [host: *Ficus* sp. 1], *P*. sp. 2 [host: *F*.* popenoei*]), with *P*. *insularis* (host: *F*. *americana*) sister to this clade, while SVDQ_ST_ and ASTRAL‐III places *P*. *herrei* (host: *F*. *paraensis*) as sister to a clade of the other three species. In addition, IQ‐TREE and SVDQ_ST_ place *Pegoscapus tonduzi* (host: *Ficus citrifolia*) sister to *Pegoscapus estherae* (host: *Ficus costaricana*) with weak support, while ASTRAL‐III places *P*. *estherae* (host: *F*. *costaricana*) sister to a large clade of fig wasps, with *P*. *tonduzi* (host: *F*. *citrifolia*) sister to them.

Phylogenetic relatedness varies for the three systems containing co‐occurring (shared and host‐specific) pollinators. The two pollinators (*P*. *hoffmeyeri* sp. A and *P*. *hoffmeyeri* sp. B) associated with *F*. *obtusifolia* are sister taxa in the phylogenies, while the three pollinator species associated with *F*. *americana* and *F*. *colubrinae* are phylogenetically distantly related (Figures 1 and 2, Figures S1 and S2). Pollinator species associated with *F*. *bullenei* and *F*. *popenoei* show phylogenetic relatedness intermediate to these two systems, where they are less divergent from one another than the pollinators associated with *F*. *americana* and *F*. *colubrinae* yet are not sister taxa within the tree. We find this result with both concatenation (Figure 1) and species tree approaches (Figure 2, Figure S2), highlighting a range of evolutionary relatedness among the systems containing co‐occurring pollinator species.

### 3.3 Population genetics

**FIGURE 3:**
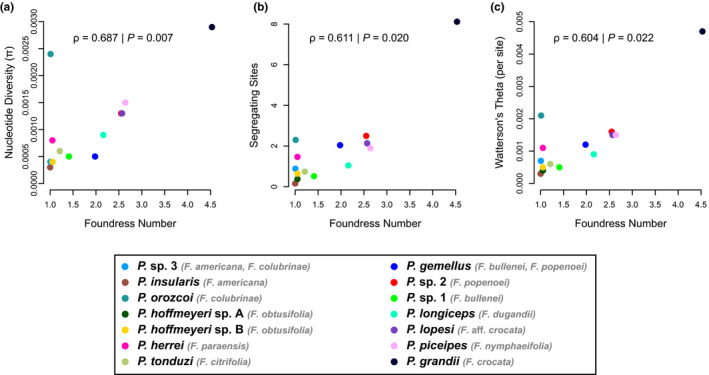
Correlation between population genetic summary statistics and average foundress number per pollinator species. Summary statistics include (a) nucleotide diversity, (b) number of segregating sites and (c) Watterson's theta (per site). The Spearman's rank correlation was used to test significance. Fourteen wasp species were included for which we had average number of foundress information (see Herre, 1989). If a wasp had multiple hosts, we used the average for the number of foundresses from the shared hosts

Population genetic statistics were variable across pollinator species, with generally wide ranges and large variances (Table 2). There is a significant positive correlation between average number of foundresses per species and genetic diversity (Figure 3). Spearman's rank correlation test recovered a statistically significant positive correlation for nucleotide diversity (*ρ* = .687, *p* = .007), number of segregating sites (*ρ* = .611, *p* = .020) and Watterson's theta (*ρ* = .604, *p* = .022) versus number of foundresses.

### 3.4 Testing for monophyly with mitochondrial DNA

Including the outgroup, we were able to capture mitochondrial DNA of the COI region from 164 individuals. Following alignment and edge trimming, these data comprised 816 base pairs of the COI mtDNA gene, with an average of 8.42 sequences (±4.51) per in‐group species. Species had on average 10.74 (±7.01) segregating sites with an average nucleotide diversity of 0.0046 (±0.0027) and an average Watterson's theta of 0.0052 (±0.0029) (Table S2). We recover a traditional barcode gap, with species averaging 0.26% intraspecific genetic divergence versus 7.67% interspecific genetic divergence (Table S2). In the gene tree, all wasp species are recovered as monophyletic with strong support, with all but one species (*Pegoscapus lopesi*, bootstrap value of 99) having a bootstrap value of 100 (Figure S3). There is, however, little support for phylogenetic relationships among species, as most nodal support values are low. Consistent with how individuals cluster within species with the nuclear data, the same clustering of individuals within species is recovered with data from the mitochondrial genome, providing strong support for what we consider a pollinator species in this community. Given the low support values for interspecific relationships, we make no comparisons between the structure of the nuclear genome phylogeny and the mitochondrial gene tree. Comparing how individuals cluster within species in the nuclear phylogeny and mitochondrial gene tree, however, these data provide no evidence of recent cytonuclear discordance.

### 3.5 Testing for admixture and gene flow

**FIGURE 4:**
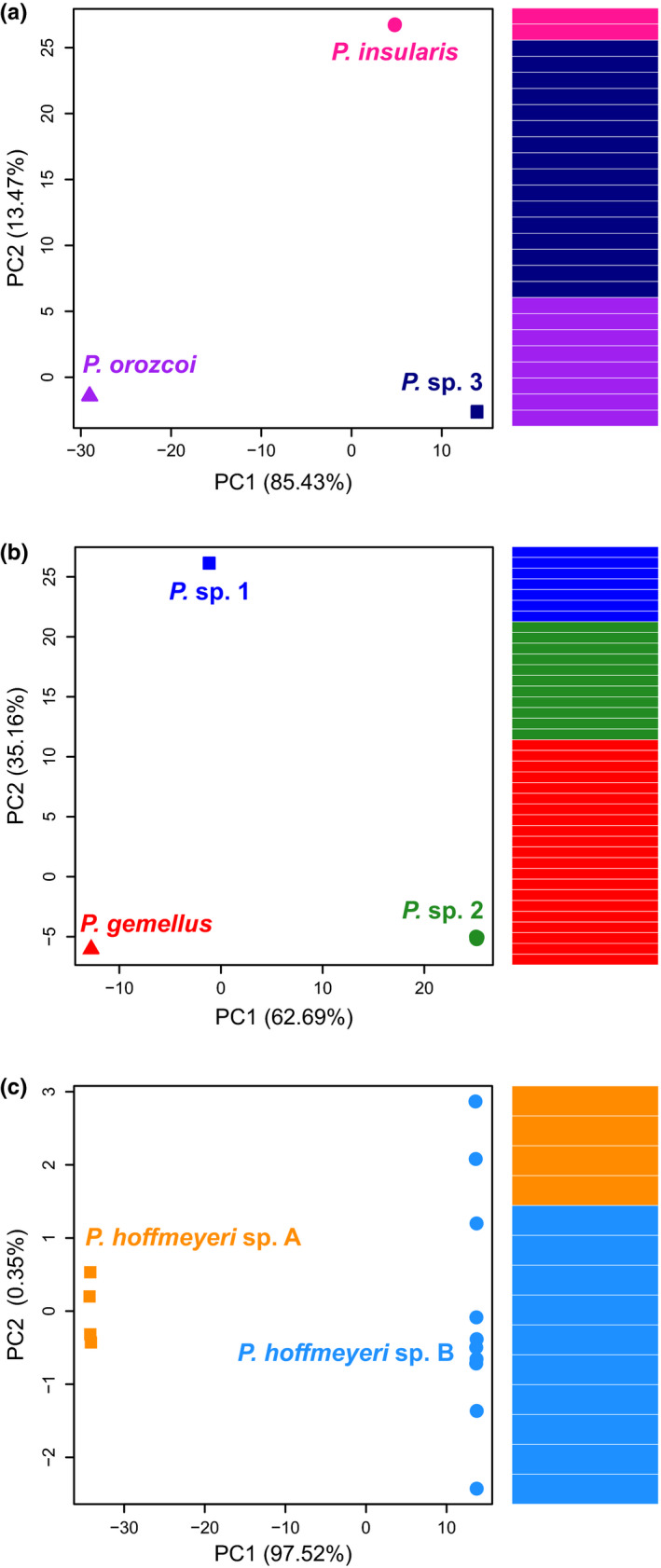
Principal components analysis (PCA) and Structure plots for the three systems where multiple pollinators interact with one or two fig species. Panel (a) represents three pollinator species associated with *Ficus americana* and *Ficus colubrinae*. Panel (b) represents three pollinator species associated with *Ficus bullenei* and *Ficus popenoei*. Panel (c) represents two pollinator species interacting with *Ficus obtusifolia*

There was no evidence of admixture or introgression in the three systems (AC, BP and O) containing co‐occurring pollinator species. Distinct clusters corresponding to species are recovered in the PCA analyses, with no signal of hybridization (Figure 4). For both AC and BP, wasp species plot in distinct PCA space with little intraspecific variance—individuals within species appear as a single point—in comparison with the much larger interspecific variance (Figure 4a,b). The two wasp species in O are well differentiated in PC1, but show some intraspecific spread in PC2, although this axis only comprises 0.35% of the variance (Figure 4c). These results were further supported in Structure, as individuals were assigned to their respective species with no evidence of admixture (Figure 4). In contrast to seeing shared ancestry indicative of admixture and introgression, each individual maps unambiguously to its respective species. Thus, in systems where opportunity for hybridization is present, we detect none.

TreeMix estimated a population graph consistent with the other phylogenetic approaches (Figure S4). Some differences were seen, mainly at places with short internal branches and low support along the backbone of the tree. Adding migration events to the tree only incrementally improved the proportion of variance explained by the model (Table 3). For example, the population graph with no admixture events explained 98.45% of the variation. Adding one hybridization event to the graph only increased the proportion of variation explained by the model to 98.62%. The minimal improvement in models including migration events suggests hybridization and introgression are not important processes in this pollinator community.

**TABLE 3:** Proportion of variation explained by the different models in TreeMix

Model	Admixture events	Percent variation
m0	0	98.45%
m1	1	98.62%
m2	2	98.76%
m3	3	98.81%
m4	4	98.90%
m5	5	98.96%

## 4 DISCUSSION

We collected genome‐wide ultraconserved element (UCE) loci from 19 pollinator wasp species associated with all 16 sympatric host strangler fig species found in the vicinity of the Panama Canal Zone. We used these data to estimate phylogenetic relationships and conduct detailed tests for hybridization and introgression among all 19 pollinator wasp species. We analysed the data within the context provided by this ecologically and evolutionarily well‐characterized strangler fig (Herre, 1989; Machado et al., 2005). Specifically, this group of pollinators has exhibited frequent host switching throughout their shared evolutionary history with their associated fig hosts (Satler et al., 2019), providing opportunities for hybridization in both host fig and pollinator wasp species. In the host figs, evidence suggests ongoing hybridization and introgression (Jackson et al., 2008; Machado et al., 2005). In the pollinator wasps, F1 pollinator hybrids have been previously detected using a combination of COI and microsatellites in one pair of intensively sampled pollinators associated with one of the hosts in this community (Molbo et al., 2003, 2004). Based on our sampling of genome‐wide UCE data, we found all 19 pollinator species to be well‐delimited genetically and show high interspecific divergence. Even among pollinator species with the potential for mating and reproducing within the same syconia of the same host fig species, we detected no evidence of hybrid individuals or introgression. In contrast to the host figs, our results suggest that hybridization and introgression play a negligible role in the evolutionary history of this community of Panamanian fig pollinator wasps.

Because reproduction for both fig and wasp occurs in the same structure (the syconium), there is considerable overlap in the conditions that facilitate hybridization in both partners in this mutualism. Opportunities for hybridization and introgression depend on physical access of heterospecific gametes (pollen and ovaries for the figs, sperm and eggs for the wasps). The conditions for successful hybridization in the host figs, however, are less restrictive. Although foundress wasps must be attracted to the floral volatiles of a different host species and enter a syconium and pollinate receptive fig inflorescences, only a single wasp from a heterospecific host need enter a fig to unite heterospecific pollen and ovules. The resulting hybrid seeds must be viable, and introgression is only possible if the hybrid seeds produce viable seedlings that survive to reproduce and backcross with one of the parental species. For example, Moe and Weiblen (2012) recovered seven hybrid individual figs (one F1, six backcrosses) among 300 trees sampled from six species of a community of New Guinea dioecious figs. Although experimental hybrid seeds germinated and grew at rates comparable with nonhybrid seeds, limited introgression in the system was attributed to low frequencies of pollinator sharing, where only six of 372 foundresses visited non‐natal host species (Moe et al., 2011).

Hybridization followed by successful introgression is strongly suggested to be a relatively frequent occurrence in the Panamanian fig hosts (Jackson et al., 2008; Machado et al., 2005). The processes of hybridization and introgression also appear to be more important for figs in general, as they have been detected in numerous fig systems (Compton, 1990; Compton et al., 2009; Cornille et al., 2012; Van Noort et al., 2013; Wang et al., 2016; Wilde et al., 2020). In particular, cytonuclear discordance between plastid and nuclear genomes provides additional support that hybridization and introgression have played an important role in shaping diversification patterns in *Ficus* (Bruun‐Lund et al., 2017; Renoult et al., 2009). Moreover, Wang, Zhang, et al. (2021) analysed whole‐genome sequence data and suggested extensive hybridization and introgression among all major lineages of *Ficus*. Thus, accumulated evidence suggests that host switching and mixed fig–pollinator associations leads to hybridization and introgression in the figs, which have been consequential in shaping the evolutionary history of this clade of plants.

Yet is the situation analogous for the pollinator wasps? One reason to question this is that the conditions for wasp hybridization would appear to be much more stringent. Two or more foundress wasps representing different species must enter the same individual receptive fig syconium. After pollination and oviposition, heterospecific male and female wasp offspring must develop to maturity and mate, the mated females must successfully disperse to a new receptive fig tree and enter a syconium, and the resulting hybrid offspring must survive, develop, reproduce and disperse. Introgression requires that hybrid wasps contribute to successful backcrosses that also must take place in a fig syconium where other foundresses from either parental species have successfully oviposited. Because this all takes place inside a fig syconium, opportunities for hybridization among pollinators associated with single‐foundress figs or nearly single‐foundress figs will be severely restricted (Table 1).

Among the pairs of Panamanian wasp species that share the same host fig species, there appears to be no clear pattern to their degree of phylogenetic relatedness. Only *P*. *hoffmeyeri* sp. A and *P*. *hoffmeyeri* sp. B associated with *F*. *obtusifolia* are recovered as sister species in our phylogeny (Figures 1 and 2, Figures S1 and S2), and these are the only two species with evidence supporting occasional F1 hybrid offspring (Molbo et al., 2003, 2004). The other co‐occurring pollinators span the phylogenetic breadth of our sampled community, with wasps associated with *F*. *bullenei* and *F*. *popenoei* being relatively closely related (but not sister taxa), while wasps associated with *F*. *americana* and *F*. *colubrinae* are distantly related (Figures 1 and 2, Figures S1 and S2). These Panamanian observations are consistent with estimates that 32.1% of co‐occurring pollinators of monoecious fig species are sister species (Yang et al., 2015). Importantly, despite previous evidence for occasional F1 hybridization events between the two *F*. *obtusifolia* pollinators (0.9%, Molbo et al., 2003, 2004), we found no evidence for F1 or backcrossed hybrids between these two species, or introgression in any of the species.

Because the number of wasp individuals we genotyped was between 2 and 21 per species, we only have modest statistical power to detect occasional first‐ or second‐generation hybridization events. For example, if the frequency of F1 hybrids between the two *F*. *obtusifolia* pollinators is 0.009 (as reported by Molbo et al., 2004), our sample size of 14 wasps would only provide an 11.9% chance of detecting one or more hybrids. At this hybrid frequency, 77 wasps would need to be assayed to have a 50% chance of sampling at least one F1 individual. Nonetheless, consider that if F1 hybrids are formed at a frequency of 0.009, and if this represents the rate of gene migration (*m*) between wasp species, then, with the large effective size of fig wasp populations (*N*e > 1000, Xun et al., 2021), the rate of effective gene flow (*N*_e_ *m*) between species would be >9 and would be expected to greatly limit differentiation (*F*_ST_ < 0.03) between species (Wright, 1949). In fact, all of the wasp species in our study are highly genetically differentiated from each other (Figures 1, 2, 3). This indicates that if undetected F1 hybrids are being formed, they are not a bridge to effective gene migration between species.

While our power to detect recent hybrid formation may be limited by the number of wasps per species that we genotyped in this study, the large number of UCE loci we assayed gives us substantial power to detect historical introgression. If hybridization has occurred in evolutionary time, resulting in even low levels of recombination between parental species genomes, with our comprehensive data set of UCE loci and the statistical analyses we have employed, we should have been able to detect that signal. We did not. Given our observations, how might different processes that affect prezygotic (host recognition andlikelihood of sharing individual fig inflorescences) and postzygotic (reproductive incompatibility between wasp species and hybrid breakdown) barriers limit hybridization and introgression among pollinator species?

Host choice by the pollinator fundamentally influences patterns of gene flow for both the fig and the wasp, and the potential for hybridization within either lineage. The degree of affinity of a particular wasp species towards particular host floral volatile blends appears to be an important prezygotic barrier limiting encounter rates of heterospecific wasps (Hossaert‐McKey et al., 2010; Wang, Yang, et al., 2021). In 17 of the 19 pollinator species sampled in the Panamanian community, wasps are predominately attracted to a single host fig species (Table 1). This high level of affinity for a particular host appears to be based on wasps recognizing consistent differences among the volatile chemical signal produced by host figs (A. Oldenbeuvingen, X. Florez, and E. A. Herre, unpublished data). Wasp species that share fig hosts, however, are often not sister species, both in this community and more broadly across *Ficus* (Yang et al., 2015). The observation of multiple, phylogenetically divergent pollinator species attracted to the same host fig raises important questions: What processes have shaped the genetics and biochemical pathways of host chemical signal production, of wasp chemosensory detection and of wasp volatile preference? Addressing this series of questions will require studies that explicitly connect host volatile chemical composition with wasp preferences (antennogram and gene expression studies) within the context of detailed phylogenetic and ecological studies, preferably across multiple sites and fig–wasp taxa.

For the host figs, effective population sizes number in the hundreds of individuals (Nason et al., 1998), and individual fig fruit crops usually bear tens of thousands of figs (Korine et al., 2000). Even if heterospecific wasps recognize the same host fig species, a precondition for pollinator hybridization is that two or more heterospecific foundress wasps oviposit successfully in the same individual fig syconium. In the Panamanian strangler fig species we studied, the average foundress numbers per syconium range between one and four‐and‐a‐half (Herre, 1989). For species with syconia pollinated almost exclusively by a single foundress (*F*. *americana* and *F*. *colubrinae*; Table 1), the opportunities for wasp hybridization will be low to nearly nonexistent. In contrast, in fig species commonly having multiple foundresses per syconium, if heterospecific wasps are attracted, then the offspring of pollinators have greater opportunities for interspecific mating. Higher mean foundress numbers will also determine wasp population structure which in turn will affect many aspects of these wasp species (sexual competition and sex ratios, heterozygosity) (Herre, 1985, 1989, 1993; Molbo et al., 2003, 2004). Notably, we found a significant positive correlation between the average number of foundresses and genetic diversity of UCE loci across pollinator species (Figure 3). Nevertheless, we detected no evidence of successful hybridization or introgression in any pollinator species, including those associated with fig hosts exhibiting higher average foundress numbers (*Ficus nymphaeifolia*, with 2.6 foundresses, or *Ficus crocata*, with 4.5 foundresses). Thus, when multiple foundresses are present, outbreeding occurs within pollinator species, but heterospecific crosses—and subsequent introgression—do not occur between pollinator species.

Several postzygotic barriers potentially play a role in restricting successful hybridization and introgression among the pollinator wasps. Greater genetic divergence usually corresponds to greater degrees of reproductive isolation (Coyne & Orr, 1989, 1997). We expect that the considerable divergence among the 19 pollinator species in this community (Figure 1) might preclude successful hybrid formation due to functional incompatibilities among either nuclear–nuclear or nuclear–cytoplasmic components derived from different parental species in the genomes of hybrid individuals. For example, the presence of diploid males—reported in some hybrid pollinators associated with *F*. *obtusifolia* (Molbo et al., 2004)—suggests a breakdown in the haplodiploid sex determination mechanism. In a mating between the males of one species and the females of another species, only the diploid F1 daughters will be hybrid offspring; haploid males develop from unfertilized eggs, so will inherit only portions from their mother's nuclear and cytoplasmic genomes and therefore will not represent genetic mixes of both parental species. If a hybrid F1 daughter is able to develop, mate, disperse and enter a new fig syconium and successfully oviposit, her F2 offspring will then almost certainly contain hybrid males with nuclear genetic components from both parental species. If the haploid recombinant genomes of these F2 hybrid males are not functionally compatible, then we may expect increased mortality and increased sterility. For example, studies of hybridization in haplodiploid *Nasonia*, another chalcidoid wasp, show significant negative effects on viability and fecundity in F2 hybrid males (Breeuwer & Werren, 1995; Gadau et al., 1999; Koevoets et al., 2012). Because co‐occurring fig wasp pollinators are usually distantly related species (Satler et al., 2019; Yang et al., 2015), these effects could be exacerbated in hybrid individuals that would contain mixes of more divergent genomes. Importantly, when F1 hybrid females are single foundress, all potential subsequent matings between their sons and daughters will be between F2 hybrids. Thus, the ability of those F2 daughters to mate will be strictly determined by the development and reproductive capability of their F2 hybrid male siblings.

An additional potential postzygotic barrier to hybridization and introgression in the wasps is *Wolbachia*. These maternally inherited cytoplasmic bacteria are known to cause drastic reductions of hybrid formation in *Nasonia* wasps (Bordenstein et al., 2001). *Wolbachia* are commonly found across insect species (Werren, 1997) and are particularly common in fig wasps (e.g. Haine & Cook, 2005; Sun et al., 2011). Pollinator and nonpollinator wasps associated with the fig community in central Panama show *Wolbachia* occurrence in 59% of wasp species (Shoemaker et al., 2002), including many of the species sampled in this study. In all fig species in which different wasp pollinators co‐occur, at least one of the pollinator species shows partial or complete *Wolbachia* infection (Shoemaker et al., 2002), raising the possibility that these bacteria influence the chances of successful hybridization. So even though hybridization has been documented in hymenopterans (e.g. Beresford et al., 2017; Feldhaar et al., 2008; Linnen & Farrell, 2007), prezygotic and postzygotic barriers specific to the fig wasp mating system provide additional barriers limiting hybridization and introgression in these insects.

Our results are based on deep genomic sampling from 19 wasp species that pollinate a complete sympatric assemblage of 16 host fig species. Although few studies explicitly test for hybridization and introgression among a community of fig wasps, our findings are consistent with results from other studies of fig wasp genetics. For example, Sutton et al. (2017) sampled three pollinator species (*Pleistodontes imperialis*; ‘species 2’, ‘species 3’ and ‘species 4’) co‐occurring with a single host fig species, *Ficus rubiginosa*. Using mitochondrial DNA and nine microsatellite loci, they detected no evidence of hybridization or introgression among the three wasp species. Sutton et al. (2017) recovered this result even though 13.38% of syconium contained multiple pollinator species, demonstrating the opportunity for wasp hybridization was present. Thus, our results showing a lack of interspecific hybridization and introgression among fig wasps are consistent with previous studies in our system (Molbo et al., 2003, 2004) and across other fig wasp systems (Sutton et al., 2017) where these questions have been addressed.

Concerning interpopulation gene flow within a single fig wasp species, a recent study by Cooper et al. (2020) recovered evidence for historical gene flow between allopatric populations of the pollinator *Pleistodontes nigriventris* associated with *Ficus watkinsiana*. Specifically, using whole genome sequencing data from four male pollinator wasps (two each from two populations) of *P*. *nigriventris*, Cooper et al. (2020) recovered support for a model containing an instantaneous admixture event ~57 kya between the two geographically distinct populations. The findings from this intraspecific study provide an informative contrast to our multispecies interspecific results. First, Cooper et al. (2020) show that migration and gene flow are processes operating within pollinator wasp species. Second, the two allopatric populations of *P*. *nigriventris* are separated by over 1000 km, reflecting the disjunct distribution of its host species, *F*. *watkinsiana*, and providing evidence for the long‐distance dispersal capability of fig wasps (Ahmed et al., 2009; Nason et al., 1998; Yu et al., 2010). Third, Cooper et al. (2020) use whole‐genome sequence data coupled with a model‐based approach to generate their inference of a historical admixture event. The power and capability of whole‐genome sequence data for revealing processes like admixture is becoming readily apparent (e.g. Taylor & Larson, 2019). Although we sampled over 1000 UCE loci spanning the genomes of pollinator species, these highly conserved gene regions may lack the sensitivity for detecting the genomic signals of certain processes, both because of their conserved nature and because they represent a small percentage of the genome. While we expect our results showing a lack of interspecific hybridization and introgression will hold with additional data, we may not be able to fully address this question without whole genome sequence data and population‐level sampling.

Many studies of the associations of pollinating wasps with their host figs tend to focus on one or a few species interactions based on genetic data collected at one or a few locales. The coupling of geographic sampling with detailed genetic characterization of figs and their pollinators, however, often reveals that multiple pollinators are associated with a single host fig across the range of the fig species (e.g. Bain et al., 2016; Darwell et al., 2014; Haine et al., 2006; Michaloud et al., 1996; Peng et al., 2008). For example, Yu et al. (2019) recovered nine parapatrically distributed pollinator species associated with *Ficus hirta* in South‐East Asia. Multiple pollinator species in allopatric and parapatric distributions and associated with the same host suggests pollinator wasps speciate in allopatry before potentially coming back into sympatry. Souto‐Vilarós et al. (2019) examined six species pairs of figs and wasps found along an elevational gradient in New Guinea and found an increased rate of genetic divergence with elevational distance among the wasps relative to their host figs. They suggest pollinators have an increased speciation rate, with populations of wasps diverging in isolation faster than their hosts, effectively decoupling speciation processes in the two mutualist taxa (Compton et al., 2009; Machado et al., 2005; Peng et al., 2008; Satler et al., 2019; Van Noort et al., 2013).

Despite the ability of pollinating wasps to disperse many kilometres (Ahmed et al., 2009; Nason et al., 1998), broad geographic sampling suggests that multiple distinct pollinator species—in allopatric and parapatric distributions—characterize many, if not most, host fig species across their ranges. Molecular data also suggest a previously underappreciated prevalence of host switching in Neotropical strangler figs, as well as figs in general (Cruaud et al., 2012; Jackson et al., 2008; Satler et al., 2019; Wang, Zhang, et al., 2021; Yang et al., 2015). We suspect that combinations of pre‐ and postzygotic barriers contribute to the reproductive isolation of pollinator wasps (Molbo et al., 2003, 2004; Satler et al., 2019). At the prezygotic level, the opportunities for interspecific matings are fairly limited due to small chances that heterospecific foundress wasps oviposit successfully in the same individual fig syconium and due to possible unknown premating barriers. At the postzygotic level, low hybrid fitness can result from intrinsic genetic incompatibilities that would limit not only the ability of hybrids to produce viable and fertile offspring, but could also affect the capacity of hybrid wasps to locate, enter, pollinate and oviposit in a receptive syconium. Furthermore, the prevalence of *Wolbachia* in fig wasps can also play a major role in postzygotic isolation. When pollinator species experience secondary contact, we suspect that, relative to their plant hosts, these barriers contribute to less fit hybrids and reinforce species boundaries (Nosil et al., 2003). We suggest that the still poorly understood processes of pollinator speciation and extinction play a more important role than hybridization and introgression in shaping evolutionary dynamics of fig pollinating wasps and their host associations in space and time. Future biogeographic, phylogenetic and population genetic analyses coupled with targeted experimental studies are needed to determine the relative importance of different mechanisms in preventing interspecific mating, hybrid production and introgression in pollinating fig wasps.

## 5 CONCLUSIONS

We have focused on detecting evidence for historical introgression across an entire Panamanian fig and wasp community with an evolutionary history of host sharing, pollinator sharing and host switching. Given the reproductive biology of fig pollinator wasps, multiple barriers appear to be important for maintaining species boundaries. Before there can be opportunity for hybridization, foundresses from different fig pollinator species need to locate, enter, and oviposit in the same fig syconium, with successful development of offspring to maturity. Because in figs foundress numbers are related to the likelihood of the offspring of different wasps encountering potential mates of other foundresses, we have sampled pollinators from hosts that ranged in number of foundresses per syconium. Nonetheless, we find no evidence of successful hybridization leading to introgression among the wasps, even in those species with high foundress numbers and shared pollinator species. This lack of hybridization and introgression strongly suggests the existence of strong reproductive isolation mechanisms in Panamanian fig pollinators. Our results suggest that, unlike their host figs, interspecific gene flow has not been important to the evolution of these fig pollinating wasps. More generally, we suggest that studies across host–pollinator mutualisms will support the suggestion that the evolutionary history of the host plants is relatively more influenced by successful hybridization and introgression than in their insect pollinators.

## AUTHOR CONTRIBUTIONS

JDS, TAH, EAH and JDN designed the study. AGZ, EAH and CAM collected the fig wasp samples. JDS generated and processed the sequence data. JDS conducted all analyses. JDS, EAH and JDN wrote the paper, and all authors contributed to revised versions of the manuscript and approved of the final version.

## Supporting information

Supplementary Material

## Data Availability

Raw sequence data are available from the NCBI Sequence Read Archive (SRA) under BioProject ID: PRJNA789240 (BioSample accessions: SAMN23703750–SAMN23703925). NCBI BioSample accession numbers for individual wasps are included in Table S1. All data sets and custom scripts are available on Dryad (https://doi.org/10.5061/dryad.fbg79cnwk). Benefits Generated: Benefits from this research accrue from the sharing of our data and results on public databases as described above.
