# Supplementary material for: Genome‐wide sequence data show no evidence of hybridization and introgression among pollinator wasps associated with a community of Panamanian strangler figs
Source: Mol Ecol. 2022 Feb 7;31(7):2106–23. doi: 10.1111/mec.16373 (PMC9545327; doi:10.1111/mec.16373)
Supplement: Supplementary file 1 — Supplementary Material [file MEC-31-2106-s001.pdf]

Supplemental Information for:

Genome-wide sequence data show no evidence of hybridization and introgression among pollinator wasps associated with a community of Panamanian strangler figs

Jordan D. Satler, Edward Allen Herre, Tracy A. Heath, Carlos A. Machado, Adalberto Gómez Zúñiga, and John D. Nason

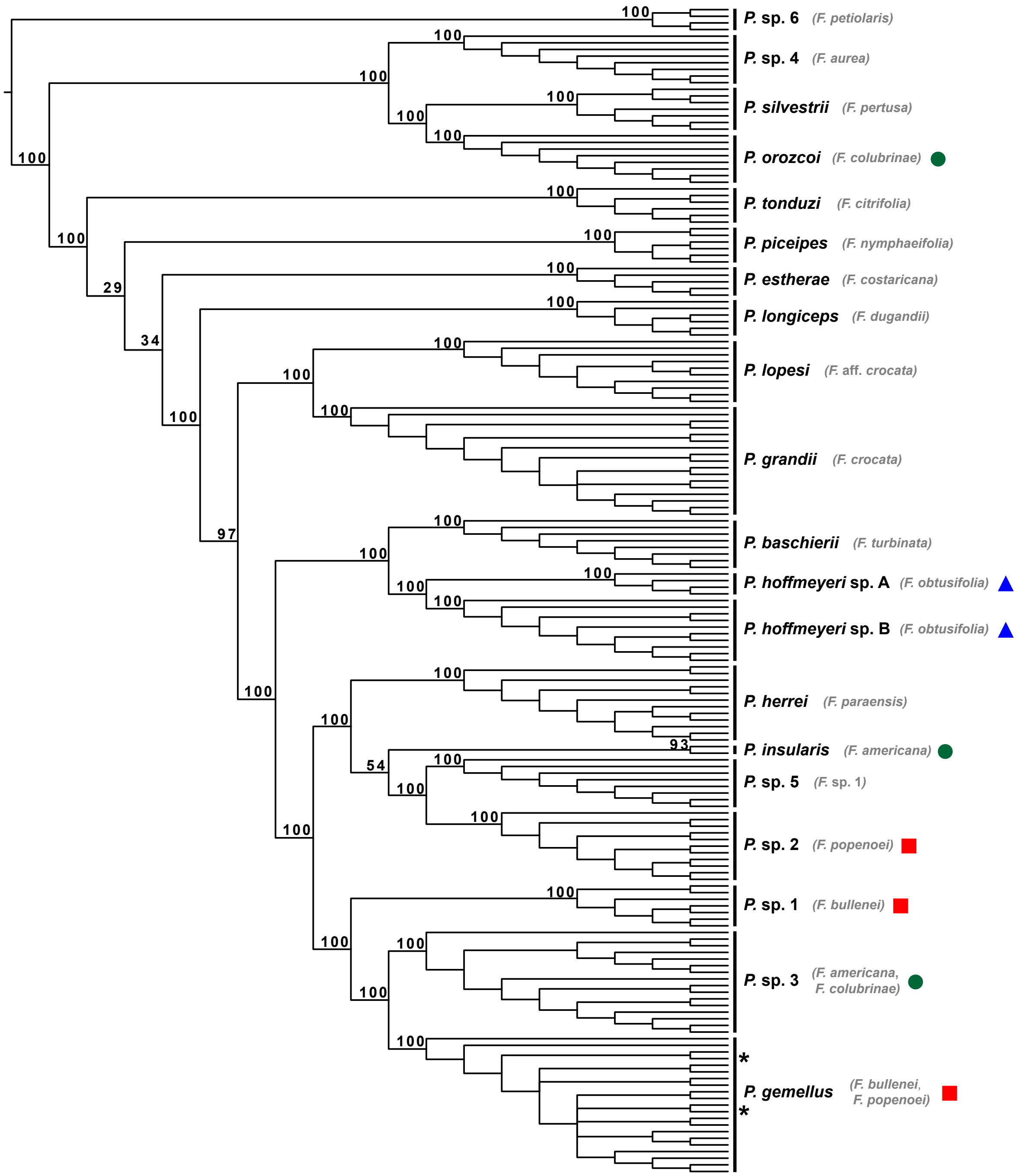

Figure S1: Lineage tree analysis with SVDquartets ($SVDQ_{LT}$) where each individual is treated as a tip in the tree. Nodal support reflecting bootstrap values are shown for species and interspecific nodes. Host fig species are displayed next to their associated wasp species. Co-occurring pollinators are denoted by a circle (green), square (red), or triangle (blue). Two individuals (represented by asterisks) of *Pegoscapus gemellus* (hosts: *Ficus bullenei*/*Ficus popenoei*) were sampled from *Ficus dugandii*, not its normal host. The undescribed pollinator associated with *Ficus petiolaris* was used to root the tree.

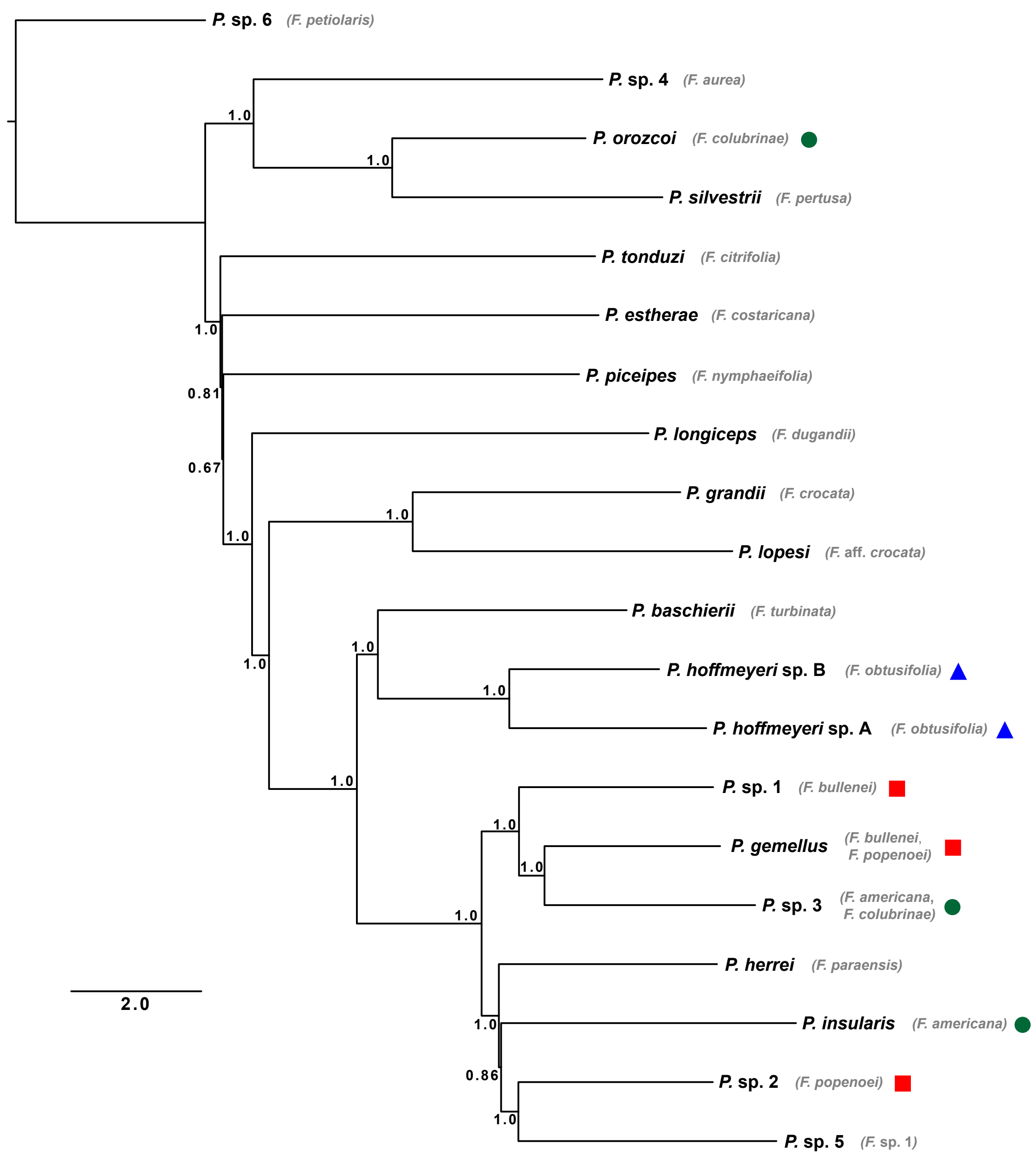

Figure S2: Species tree analysis with ASTRAL-III. Nodal support values represent local posterior probabilities. Host fig species are displayed next to their associated wasp species. Branch lengths are in coalescent units. Co-occurring pollinators are denoted by a circle (green), square (red), or triangle (blue). The undescribed pollinator associated with *Ficus petiolaris* was used to root the tree.

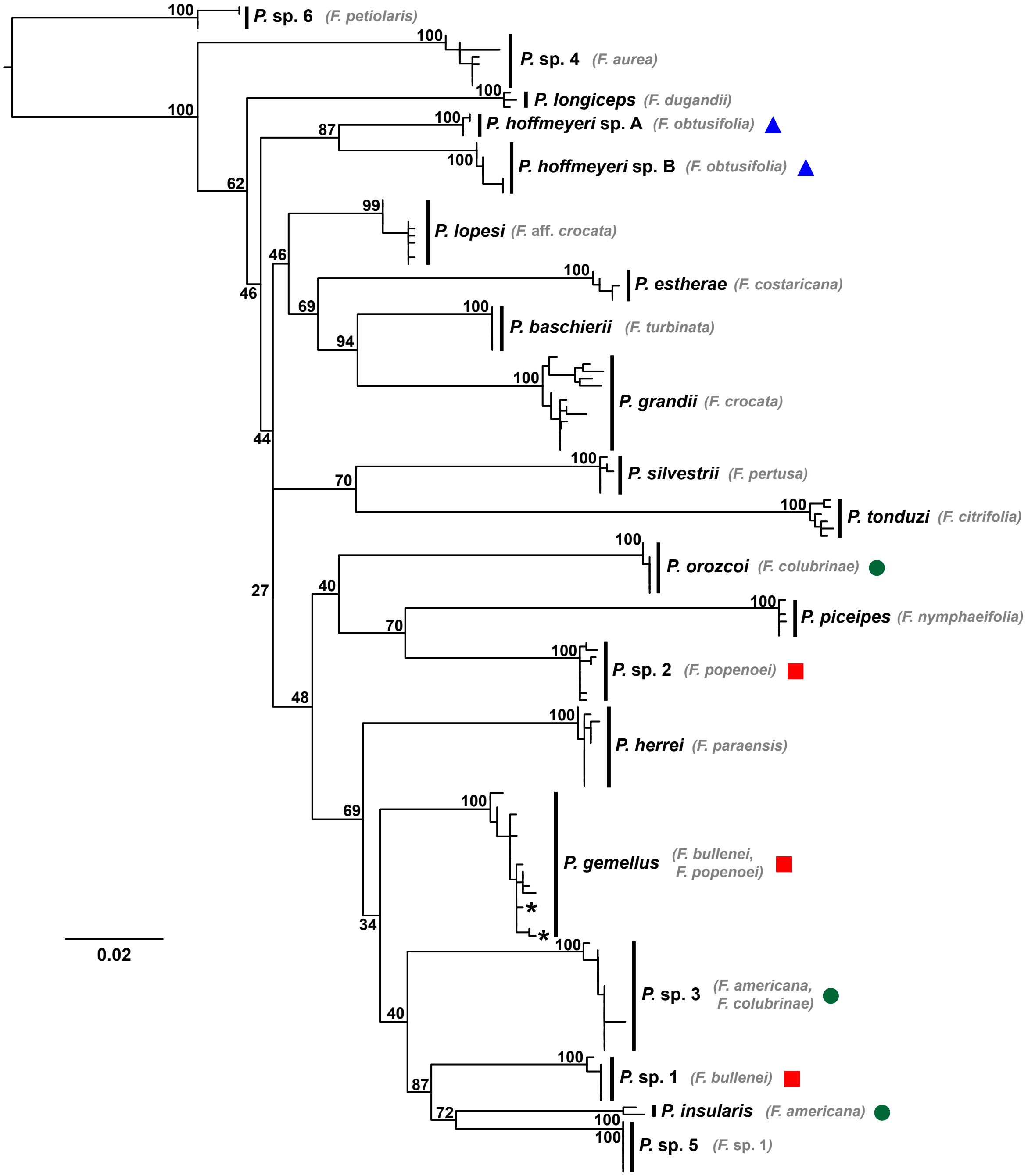

Figure S3: Maximum likelihood gene tree representing relationships among mitochondrial haplotypes. Nodal support reflecting bootstrap values are shown for species and interspecific nodes. Host fig species are displayed next to their associated wasp species. Co-occurring pollinators are denoted by a circle (green), square (red), or triangle (blue). Two individuals (represented by asterisks) of *Pegoscapus gemellus* (hosts: *Ficus bullenei*/*Ficus popenoei*) were sampled from *Ficus dugandii*, not its normal host. The undescribed pollinator associated with *Ficus petiolaris* was used to root the tree.

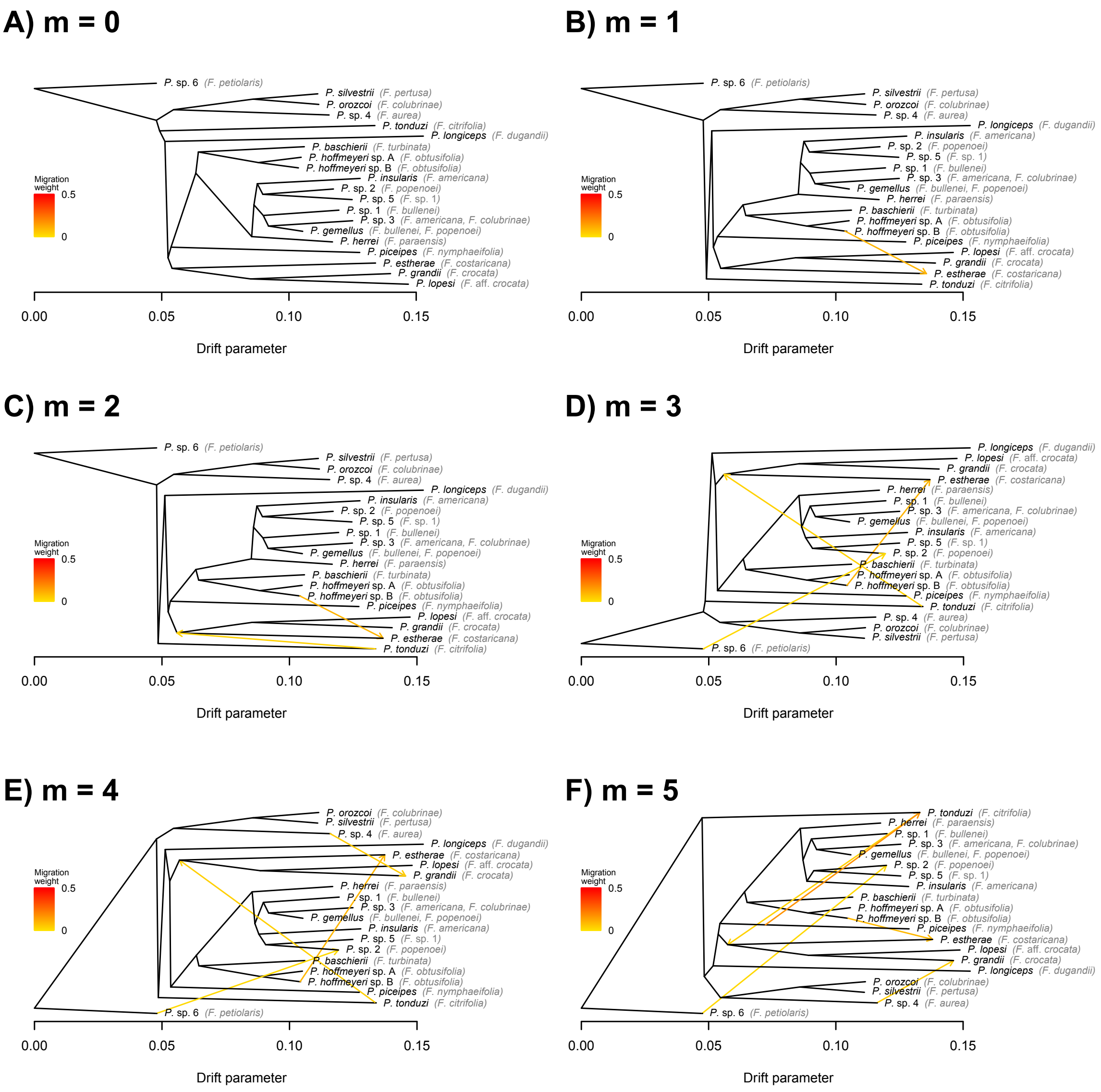

Figure S4: TreeMix results. Results show population graphs without admixture (A), and with between 1 and 5 admixture (m) events (B–F). A model without hybridization (A) is best supported by the data (Table 3). The undescribed pollinator associated with *Ficus petiolaris* was used to root the tree.

Pollinator Wasp	Individual	Sampled Host Fig	BioSample
Pegoscapus baschierii	FW403	*Ficus turbinata*	SAMN23703750
Pegoscapus baschierii	FW404	*Ficus turbinata*	SAMN23703751
Pegoscapus baschierii	FW405	*Ficus turbinata*	SAMN23703752
Pegoscapus baschierii	FW406	*Ficus turbinata*	SAMN23703753
Pegoscapus baschierii	FW407	*Ficus turbinata*	SAMN23703754
Pegoscapus baschierii	FW408	*Ficus turbinata*	SAMN23703755
Pegoscapus baschierii	FW409	*Ficus turbinata*	SAMN23703756
Pegoscapus baschierii	FW410	*Ficus turbinata*	SAMN23703757
Pegoscapus estherae	FW252	*Ficus costaricana*	SAMN23703758
Pegoscapus estherae	FW253	*Ficus costaricana*	SAMN23703759
Pegoscapus estherae	FW254	*Ficus costaricana*	SAMN23703760
Pegoscapus estherae	FW255	*Ficus costaricana*	SAMN23703761
Pegoscapus estherae	FW256	*Ficus costaricana*	SAMN23703762
Pegoscapus gemellus	FW10	*Ficus bullenei*	SAMN23703763
Pegoscapus gemellus	FW283	*Ficus bullenei*	SAMN23703764
Pegoscapus gemellus	FW284	*Ficus bullenei*	SAMN23703765
Pegoscapus gemellus	FW285	*Ficus bullenei*	SAMN23703766
Pegoscapus gemellus	FW286	*Ficus bullenei*	SAMN23703767
Pegoscapus gemellus	FW287	*Ficus bullenei*	SAMN23703768
Pegoscapus gemellus	FW288	*Ficus bullenei*	SAMN23703769
Pegoscapus gemellus	FW289	*Ficus bullenei*	SAMN23703770
Pegoscapus gemellus	FW290	*Ficus bullenei*	SAMN23703771
Pegoscapus gemellus	FW307	*Ficus popenoei*	SAMN23703772
Pegoscapus gemellus	FW309	*Ficus popenoei*	SAMN23703773
Pegoscapus gemellus	FW313	*Ficus popenoei*	SAMN23703774
Pegoscapus gemellus	FW314	*Ficus popenoei*	SAMN23703775
Pegoscapus gemellus	FW360	*Ficus popenoei*	SAMN23703776
Pegoscapus gemellus	FW362	*Ficus popenoei*	SAMN23703777
Pegoscapus gemellus	FW363	*Ficus popenoei*	SAMN23703778
Pegoscapus gemellus	FW364	*Ficus popenoei*	SAMN23703779
Pegoscapus gemellus	FW367	*Ficus popenoei*	SAMN23703780
Pegoscapus gemellus	FW369	*Ficus popenoei*	SAMN23703781
Pegoscapus gemellus	W12M1	*Ficus dugandii*	SAMN23703782
Pegoscapus gemellus	W12M7	*Ficus dugandii*	SAMN23703783
Pegoscapus grandii	FW195	*Ficus crocata* X aff. *crocata*	SAMN23703784
Pegoscapus grandii	FW200	*Ficus crocata* X aff. *crocata*	SAMN23703785
Pegoscapus grandii	FW201	*Ficus crocata* X aff. *crocata*	SAMN23703786
Pegoscapus grandii	FW227	*Ficus crocata*	SAMN23703787
Pegoscapus grandii	FW228	*Ficus crocata*	SAMN23703788
Pegoscapus grandii	FW229	*Ficus crocata*	SAMN23703789
Pegoscapus grandii	FW395	*Ficus crocata* X aff. *crocata*	SAMN23703790

Pegoscapus grandii	FW396	*Ficus crocata* X aff. *crocata*	SAMN23703791
Pegoscapus grandii	FW397	*Ficus crocata* X aff. *crocata*	SAMN23703792
Pegoscapus grandii	FW398	*Ficus crocata* X aff. *crocata*	SAMN23703793
Pegoscapus grandii	FW399	*Ficus crocata* X aff. *crocata*	SAMN23703794
Pegoscapus grandii	FW400	*Ficus crocata* X aff. *crocata*	SAMN23703795
Pegoscapus grandii	FW402	*Ficus crocata* X aff. *crocata*	SAMN23703796
Pegoscapus grandii	FW435	*Ficus crocata*	SAMN23703797
Pegoscapus grandii	FW437	*Ficus crocata*	SAMN23703798
Pegoscapus grandii	FW505	*Ficus crocata*	SAMN23703799
Pegoscapus grandii	FW506	*Ficus crocata*	SAMN23703800
Pegoscapus herrei	FW423	*Ficus paraensis*	SAMN23703801
Pegoscapus herrei	FW424	*Ficus paraensis*	SAMN23703802
Pegoscapus herrei	FW425	*Ficus paraensis*	SAMN23703803
Pegoscapus herrei	FW426	*Ficus paraensis*	SAMN23703804
Pegoscapus herrei	FW551	*Ficus paraensis*	SAMN23703805
Pegoscapus herrei	FW552	*Ficus paraensis*	SAMN23703806
Pegoscapus herrei	FW553	*Ficus paraensis*	SAMN23703807
Pegoscapus herrei	FW554	*Ficus paraensis*	SAMN23703808
Pegoscapus herrei	FW555	*Ficus paraensis*	SAMN23703809
Pegoscapus herrei	FW556	*Ficus paraensis*	SAMN23703810
Pegoscapus herrei	FW557	*Ficus paraensis*	SAMN23703811
Pegoscapus herrei	FW558	*Ficus paraensis*	SAMN23703812
Pegoscapus hoffmeyeri sp. A	FW231	*Ficus obtusifolia*	SAMN23703813
Pegoscapus hoffmeyeri sp. A	FW371	*Ficus obtusifolia*	SAMN23703814
Pegoscapus hoffmeyeri sp. A	FW373	*Ficus obtusifolia*	SAMN23703815
Pegoscapus hoffmeyeri sp. A	FW378	*Ficus obtusifolia*	SAMN23703816
Pegoscapus hoffmeyeri sp. B	FW232	*Ficus obtusifolia*	SAMN23703817
Pegoscapus hoffmeyeri sp. B	FW372	*Ficus obtusifolia*	SAMN23703818
Pegoscapus hoffmeyeri sp. B	FW374	*Ficus obtusifolia*	SAMN23703819
Pegoscapus hoffmeyeri sp. B	FW375	*Ficus obtusifolia*	SAMN23703820
Pegoscapus hoffmeyeri sp. B	FW376	*Ficus obtusifolia*	SAMN23703821
Pegoscapus hoffmeyeri sp. B	FW377	*Ficus obtusifolia*	SAMN23703822
Pegoscapus hoffmeyeri sp. B	FW387	*Ficus obtusifolia*	SAMN23703823
Pegoscapus hoffmeyeri sp. B	FW388	*Ficus obtusifolia*	SAMN23703824
Pegoscapus hoffmeyeri sp. B	FW389	*Ficus obtusifolia*	SAMN23703825
Pegoscapus hoffmeyeri sp. B	FW390	*Ficus obtusifolia*	SAMN23703826
Pegoscapus insularis	FW13	*Ficus americana*	SAMN23703827
Pegoscapus insularis	FW598	*Ficus americana*	SAMN23703828
Pegoscapus longiceps	FW164	*Ficus dugandii*	SAMN23703829
Pegoscapus longiceps	FW165	*Ficus dugandii*	SAMN23703830
Pegoscapus longiceps	FW166	*Ficus dugandii*	SAMN23703831
Pegoscapus longiceps	FW167	*Ficus dugandii*	SAMN23703832
Pegoscapus longiceps	FW168	*Ficus dugandii*	SAMN23703833

Pegoscapus longiceps	FW170	*Ficus dugandii*	SAMN23703834
Pegoscapus lopesi	FW196	*Ficus crocata* X aff. *crocata*	SAMN23703835
Pegoscapus lopesi	FW198	*Ficus crocata* X aff. *crocata*	SAMN23703836
Pegoscapus lopesi	FW199	*Ficus crocata* X aff. *crocata*	SAMN23703837
Pegoscapus lopesi	FW401	*Ficus crocata* X aff. *crocata*	SAMN23703838
Pegoscapus lopesi	FW427	*Ficus* aff. *crocata*	SAMN23703839
Pegoscapus lopesi	FW428	*Ficus* aff. *crocata*	SAMN23703840
Pegoscapus lopesi	FW429	*Ficus* aff. *crocata*	SAMN23703841
Pegoscapus lopesi	FW430	*Ficus* aff. *crocata*	SAMN23703842
Pegoscapus lopesi	FW433	*Ficus* aff. *crocata*	SAMN23703843
Pegoscapus lopesi	FW434	*Ficus* aff. *crocata*	SAMN23703844
Pegoscapus orozcoi	FW544	*Ficus colubrinae*	SAMN23703845
Pegoscapus orozcoi	FW545	*Ficus colubrinae*	SAMN23703846
Pegoscapus orozcoi	FW546	*Ficus colubrinae*	SAMN23703847
Pegoscapus orozcoi	FW547	*Ficus colubrinae*	SAMN23703848
Pegoscapus orozcoi	FW548	*Ficus colubrinae*	SAMN23703849
Pegoscapus orozcoi	FW549	*Ficus colubrinae*	SAMN23703850
Pegoscapus orozcoi	FW550	*Ficus colubrinae*	SAMN23703851
Pegoscapus orozcoi	FW568	*Ficus colubrinae*	SAMN23703852
Pegoscapus piceipes	FW419	*Ficus nymphaeifolia*	SAMN23703853
Pegoscapus piceipes	FW420	*Ficus nymphaeifolia*	SAMN23703854
Pegoscapus piceipes	FW421	*Ficus nymphaeifolia*	SAMN23703855
Pegoscapus piceipes	FW422	*Ficus nymphaeifolia*	SAMN23703856
Pegoscapus piceipes	FW571	*Ficus nymphaeifolia*	SAMN23703857
Pegoscapus piceipes	FW572	*Ficus nymphaeifolia*	SAMN23703858
Pegoscapus silvestrii	FW180	*Ficus pertusa*	SAMN23703859
Pegoscapus silvestrii	FW181	*Ficus pertusa*	SAMN23703860
Pegoscapus silvestrii	FW182	*Ficus pertusa*	SAMN23703861
Pegoscapus silvestrii	FW183	*Ficus pertusa*	SAMN23703862
Pegoscapus silvestrii	FW184	*Ficus pertusa*	SAMN23703863
Pegoscapus silvestrii	FW185	*Ficus pertusa*	SAMN23703864
Pegoscapus silvestrii	FW186	*Ficus pertusa*	SAMN23703865
Pegoscapus sp. 1	FW559	*Ficus bullenei*	SAMN23703866
Pegoscapus sp. 1	FW560	*Ficus bullenei*	SAMN23703867
Pegoscapus sp. 1	FW561	*Ficus bullenei*	SAMN23703868
Pegoscapus sp. 1	FW562	*Ficus bullenei*	SAMN23703869
Pegoscapus sp. 1	FW563	*Ficus bullenei*	SAMN23703870
Pegoscapus sp. 1	FW564	*Ficus bullenei*	SAMN23703871
Pegoscapus sp. 1	FW566	*Ficus bullenei*	SAMN23703872
Pegoscapus sp. 2	FW308	*Ficus popenoei*	SAMN23703873
Pegoscapus sp. 2	FW310	*Ficus popenoei*	SAMN23703874
Pegoscapus sp. 2	FW311	*Ficus popenoei*	SAMN23703875
Pegoscapus sp. 2	FW312	*Ficus popenoei*	SAMN23703876

Pegoscapus sp. 2	FW315	*Ficus popenoei*	SAMN23703877
Pegoscapus sp. 2	FW316	*Ficus popenoei*	SAMN23703878
Pegoscapus sp. 2	FW317	*Ficus popenoei*	SAMN23703879
Pegoscapus sp. 2	FW318	*Ficus popenoei*	SAMN23703880
Pegoscapus sp. 2	FW319	*Ficus popenoei*	SAMN23703881
Pegoscapus sp. 2	FW321	*Ficus popenoei*	SAMN23703882
Pegoscapus sp. 2	FW322	*Ficus popenoei*	SAMN23703883
Pegoscapus sp. 3	FW301	*Ficus americana*	SAMN23703884
Pegoscapus sp. 3	FW327	*Ficus americana*	SAMN23703885
Pegoscapus sp. 3	FW332	*Ficus americana*	SAMN23703886
Pegoscapus sp. 3	FW339	*Ficus americana*	SAMN23703887
Pegoscapus sp. 3	FW342	*Ficus americana*	SAMN23703888
Pegoscapus sp. 3	FW350	*Ficus americana*	SAMN23703889
Pegoscapus sp. 3	FW355	*Ficus americana*	SAMN23703890
Pegoscapus sp. 3	FW443	*Ficus colubrinae*	SAMN23703891
Pegoscapus sp. 3	FW444	*Ficus colubrinae*	SAMN23703892
Pegoscapus sp. 3	FW445	*Ficus colubrinae*	SAMN23703893
Pegoscapus sp. 3	FW446	*Ficus colubrinae*	SAMN23703894
Pegoscapus sp. 3	FW447	*Ficus colubrinae*	SAMN23703895
Pegoscapus sp. 3	FW448	*Ficus colubrinae*	SAMN23703896
Pegoscapus sp. 3	FW449	*Ficus colubrinae*	SAMN23703897
Pegoscapus sp. 3	FW450	*Ficus colubrinae*	SAMN23703898
Pegoscapus sp. 3	W17D	*Ficus americana*	SAMN23703899
Pegoscapus sp. 4	FW156	*Ficus aurea*	SAMN23703900
Pegoscapus sp. 4	FW157	*Ficus aurea*	SAMN23703901
Pegoscapus sp. 4	FW158	*Ficus aurea*	SAMN23703902
Pegoscapus sp. 4	FW159	*Ficus aurea*	SAMN23703903
Pegoscapus sp. 4	FW161	*Ficus aurea*	SAMN23703904
Pegoscapus sp. 4	FW162	*Ficus aurea*	SAMN23703905
Pegoscapus sp. 4	FW591	*Ficus aurea*	SAMN23703906
Pegoscapus sp. 4	FW592	*Ficus aurea*	SAMN23703907
Pegoscapus sp. 5	FW172	*Ficus* sp. 1	SAMN23703908
Pegoscapus sp. 5	FW173	*Ficus* sp. 1	SAMN23703909
Pegoscapus sp. 5	FW174	*Ficus* sp. 1	SAMN23703910
Pegoscapus sp. 5	FW175	*Ficus* sp. 1	SAMN23703911
Pegoscapus sp. 5	FW176	*Ficus* sp. 1	SAMN23703912
Pegoscapus sp. 5	FW177	*Ficus* sp. 1	SAMN23703913
Pegoscapus sp. 5	FW593	*Ficus* sp. 1	SAMN23703914
Pegoscapus sp. 5	FW594	*Ficus* sp. 1	SAMN23703915
Pegoscapus sp. 6	FW267	*Ficus petiolaris*	SAMN23703916
Pegoscapus sp. 6	FW268	*Ficus petiolaris*	SAMN23703917
Pegoscapus sp. 6	FW269	*Ficus petiolaris*	SAMN23703918
Pegoscapus sp. 6	FW270	*Ficus petiolaris*	SAMN23703919

Pegoscapus tonduzi	FW507	*Ficus citrifolia*	SAMN23703920
Pegoscapus tonduzi	FW508	*Ficus citrifolia*	SAMN23703921
Pegoscapus tonduzi	FW509	*Ficus citrifolia*	SAMN23703922
Pegoscapus tonduzi	FW510	*Ficus citrifolia*	SAMN23703923
Pegoscapus tonduzi	FW511	*Ficus citrifolia*	SAMN23703924
Pegoscapus tonduzi	FW512	*Ficus citrifolia*	SAMN23703925

Table S1: Pollinator wasp sampling. Information includes pollinator wasp species, individual wasp sample code, sampled host fig species, and NCBI BioSample accession number.

Pollinator Wasp	Host Fig	N	π	SS	θ_w	Intraspecific (%)	Interspecific (%)
Pegoscapus gemellus	*Ficus bullenei* & *Ficus popenoei*	21	0.0062	22	0.0075	0.34 (0.00–1.00)	6.55 (4.08–11.50)
Pegoscapus sp. 1	*Ficus bullenei*	7	0.0031	8	0.0040	0.11 (0.00–0.40)	6.69 (4.06–10.80)
Pegoscapus sp. 2	*Ficus popenoei*	9	0.0056	16	0.0072	0.21 (0.00–0.62)	7.62 (5.14–12.00)
Pegoscapus sp. 3	*Ficus americana* & *Ficus colubrinae*	16	0.0035	16	0.0059	0.18 (0.00–0.94)	7.21 (4.08–12.21)
Pegoscapus insularis	*Ficus americana*	2	0.0061	5	0.0061	0.62 (0.62–0.62)	7.52 (4.77–11.39)
Pegoscapus orozcoi	*Ficus colubrinae*	8	0.0039	12	0.0057	0.05 (0.00–0.13)	8.09 (5.98–12.22)
Pegoscapus hoffmeyeri sp. A	*Ficus obtusifolia*	4	0.0014	2	0.0013	0.08 (0.00–0.12)	6.85 (4.22–10.75)
Pegoscapus hoffmeyeri sp. B	*Ficus obtusifolia*	8	0.0055	6	0.0028	0.30 (0.00–0.72)	7.45 (4.22–11.98)
Pegoscapus baschierii	*Ficus turbinata*	7	0.0010	3	0.0015	0.00 (0.00–0.00)	6.83 (4.33–11.00)
Pegoscapus estherae	*Ficus costaricana*	5	0.0039	7	0.0041	0.25 (0.00–0.49)	8.01 (5.99–11.54)
Pegoscapus grandii	*Ficus crocata*	14	0.0103	18	0.0069	0.77 (0.00–1.75)	8.00 (4.33–12.36)
Pegoscapus herrei	*Ficus paraensis*	12	0.0015	5	0.0020	0.13 (0.00–0.50)	7.46 (4.78–12.08)
Pegoscapus longiceps	*Ficus dugandii*	3	0.0033	4	0.0033	0.33 (0.25–0.37)	7.93 (5.67–11.14)
Pegoscapus lopesi	*Ficus* aff. *crocata*	10	0.0040	12	0.0052	0.29 (0.00–0.62)	6.69 (4.44–10.86)
Pegoscapus piceipes	*Ficus nymphaeifolia*	6	0.0078	17	0.0091	0.12 (0.00–0.28)	9.15 (7.26–12.46)
Pegoscapus silvestrii	*Ficus pertusa*	6	0.0027	6	0.0032	0.15 (0.00–0.37)	8.24 (5.88–10.69)
Pegoscapus tonduzi	*Ficus citrifolia*	6	0.0072	15	0.0081	0.56 (0.25–0.87)	10.75 (8.73–12.46)
Pegoscapus sp. 4	*Ficus aurea*	8	0.0095	27	0.0128	0.40 (0.00–1.02)	7.97 (4.87–11.33)
Pegoscapus sp. 5	*Ficus* sp. 1	8	0.0008	3	0.0014	0.00 (0.00–0.00)	6.72 (4.06–10.86)

Table S2: Population genetic summary statistics of the mitochondrial DNA for the pollinator wasp species. Represented are the number of mtDNA sequences (N), nucleotide diversity (π), number of segregating sites (SS), Watterson's theta per site (θ_w), and average intraspecific genetic divergence (minimum–maximum) and average interspecific genetic divergence (minimum–maximum). For genetic divergence calculations, we used a GTR model to correct for genetic distance, and excluded sequences with greater than 50% missing data. Lines separate the three host-sharing systems from the remaining species (with one-to-one fig–wasp association) found in this community.
